# Synthesis, Structural Investigations, DNA/BSA Interactions, Molecular Docking Studies, and Anticancer Activity of a New 1,4-Disubstituted 1,2,3-Triazole Derivative

**DOI:** 10.1021/acsomega.3c03355

**Published:** 2023-08-25

**Authors:** Tolga Göktürk, Esin Sakallı Çetin, Tuncer Hökelek, Hanife Pekel, Özge Şensoy, Ebru Nur Aksu, Ramazan Güp

**Affiliations:** † Department of Chemistry, Muğla Sıtkı Koçman University, 48000 Muğla, Türkiye; ‡ Department of Medical Biology, Muğla Sıtkı Koçman University, 48000 Muğla, Türkiye; § Department of Physics, Hacettepe University, 06800 Ankara, Türkiye; ∥ Department of Pharmacy Services, Vocational School of Health Services, Istanbul Medipol University, 34810 Istanbul, Türkiye; ⊥ Department of Computer Engineering, Istanbul Medipol University, 34000 Istanbul, Türkiye

## Abstract

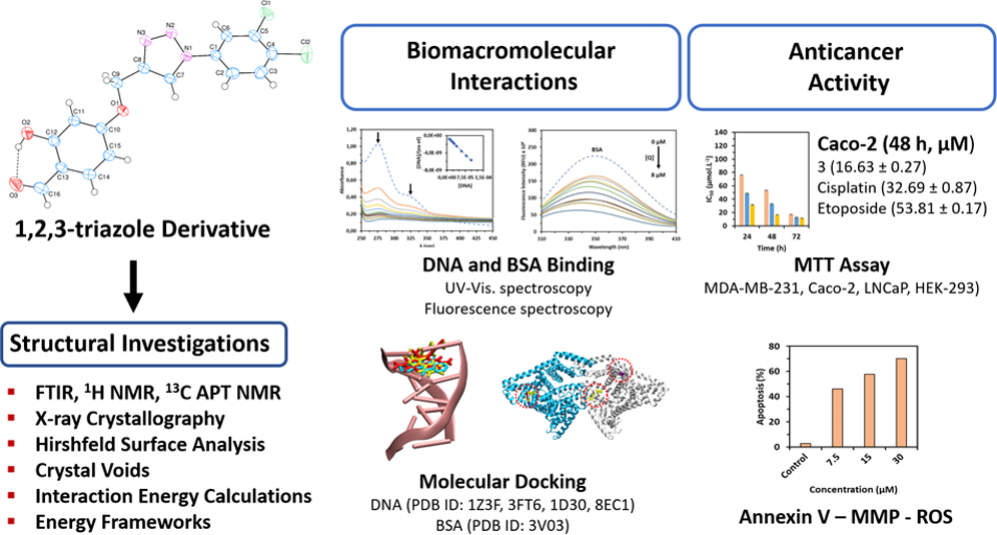

We report herein a new 1,2,3-triazole derivative, namely, 4-((1-(3,4-dichlorophenyl)-1*H*-1,2,3-triazol-4-yl)methoxy)-2-hydroxybenzaldehyde, which was synthesized by copper(I)-catalyzed azide–alkyne cycloaddition (CuAAC). The structure of the compound was analyzed using Fourier transform infrared spectroscopy (FTIR), ^1^H NMR, ^13^C NMR, UV–vis, and elemental analyses. Moreover, X-ray crystallography studies demonstrated that the compound adapted a monoclinic crystal system with the *P*2_1_/*c* space group. The dominant interactions formed in the crystal packing were found to be hydrogen bonding and van der Waals interactions according to Hirshfeld surface (HS) analysis. The volume of the crystal voids and the percentage of free spaces in the unit cell were calculated as 152.10 Å^3^ and 9.80%, respectively. The evaluation of energy frameworks showed that stabilization of the compound was dominated by dispersion energy contributions. Both in vitro and in silico investigations on the DNA/bovine serum albumin (BSA) binding activity of the compound showed that the CT-DNA binding activity of the compound was mediated via intercalation and BSA binding activity was mediated via both polar and hydrophobic interactions. The anticancer activity of the compound was also tested by the 3-(4,5-dimethylthiazol-2-yl)-2,5-diphenyltetrazolium bromide (MTT) assay using human cell lines including MDA-MB-231, LNCaP, Caco-2, and HEK-293. The compound exhibited more cytotoxic activity than cisplatin and etoposide on Caco-2 cancer cell lines with an IC_50_ value of 16.63 ± 0.27 μM after 48 h. Annexin V suggests the induction of cell death by apoptosis. Compound **3** significantly increased the loss of mitochondrial membrane potential (MMP) levels in Caco-2 cells, and the reactive oxygen species (ROS) assay proved that compound **3** could induce apoptosis by ROS generation.

## 1 Introduction

Cancer is one of the most common diseases in the world, causing the deaths of millions of people every year.^1^ For this reason, the investigation of anticancer compounds has emerged as an essential field in medicinal chemistry.^2,3^ The therapeutic compounds that can bind to DNA and proteins and exhibit anticancer activity have attracted a significant amount of attention.^4,5^ Among these, triazoles are well-known heterocyclic organic compounds that are associated with a variety of biological activities. These significant biological and medicinal properties of triazoles have encouraged investigations into the synthesis and detailed structural characterization of new 1,2,3-triazole derivatives.^6,7^ The Cu(I)-catalyzed azide–alkyne cycloaddition, also known as the CuAAC “click reaction,” significantly improved the preparation of 1,4-disubstituted 1,2,3-triazole-based compounds under mild conditions.^8,9^

Especially, 1,2,3-triazole derivatives have been thoroughly studied as potent tools in anticancer research.^10−12^ A few triazole compounds have also been transferred to clinical trials. Among them, tazobactam, which is a non-nucleoside inhibitor of reverse transcriptase activity,^13^ β-lactamase,^14^ carboxyamidotriazole (CAI), which are signal transduction blockers,^15^ letrozole, and anastrozole, which are triazole derivative drugs targeting aromatase, are used to treat breast cancer.^15^

An effective interaction between a small drug molecule and DNA or proteins typically dictates its anticancer activity. For this reason, it is critical to examine binding interactions between DNA and anticancer compounds in order to find effective anticancer drugs.^16−18^ Anticancer drugs bind to DNA mainly in three modes: electrostatic attraction, groove binding, and intercalation.^19^ However, anticancer drugs interact with DNA in a variety of complex ways, the exact mechanisms of which are still unknown and need to be deeply investigated. In these circumstances, research into how compounds interact with DNA is critical for the design and development of anticancer drugs that are effective against a wide range of diseases.

In addition to DNA, proteins have also attracted the attention of researchers as prime molecular targets. Albumins are the most important proteins found in blood plasma, transporting both endogenous and exogenous compounds.^20^ The investigation of the drug–protein interactions is also critical, since most of the drugs, which are bound to serum albumin, are transported as drug–protein adducts.^21^

Molecular docking is one of the most popular techniques used for examining the molecular mechanisms of interactions formed between small compounds and their potential biomacromoleculer targets including DNA or bovine serum albumin. The use of molecular docking to identify preferred sites for binding and the optimum orientation of drug candidates on a target protein can be helpful when designing chosen analogues with higher activities, as this information reveals the binding affinity and potential activity of the drug candidate.^22−24^

Despite the market’s abundance of anticancer medications and the fact that many are currently undergoing clinical trials, there is an urgent need for the development of effective, targeted, less toxic anticancer drugs to treat the millions of new cases of cancer diagnosed each year.

Therefore, in light of these facts, we have decided to design a novel molecule that can be used as an anticancer agent. We used click chemistry to synthesize 4-((1-(3,4-dichlorophenyl)-1*H*-1,2,3-triazol-4-yl)methoxy)-2-hydroxybenzaldehyde as a new 1,4-disubstituted 1,2,3-triazole derivative. The compound was characterized using elemental analyses, Fourier transform infrared spectroscopy (FTIR), ^1^H and ^13^C APT NMR, and single-crystal X-ray diffraction (SCXRD). The HS analysis, interaction energy and energy framework investigations, and the volume of the crystal voids of the compound were also performed. Additionally, DNA and bovine serum albumin interaction properties of the synthesized compound were further studied by applying in vitro and in silico methods. Finally, its anticancer activity was evaluated using a 3-(4,5-dimethylthiazol-2-yl)-2,5-diphenyltetrazolium bromide (MTT) assay against cancer cell lines including MDA-MB-231 (breast cancer), LNCaP (prostate carcinoma), Caco-2 (colorectal adenocarcinoma), and HEK-293 (the normal cell line). Annexin V, mitochondrial membrane potential (MMP), and reactive oxygen species (ROS) assays were also carried out.

## 2 Results and Discussion

### 2.1 Synthesis and Characterization

**Scheme 1:**
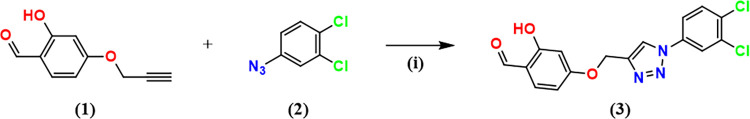
Synthesis Route of the 1,2,3-Triazol Derivative. (i) CuAc/NaAsc, CH_2_CI_2_/H_2_O, 8 h, RT

4-((1-(3,4-Dichlorophenyl)-1*H*-1,2,3-triazol-4-yl)methoxy)-2-hydroxybenzaldehyde (**3**) was synthesized by the copper(I)-catalyzed azide–alkyne cycloaddition (CuAAC) reaction between 2-hydroxy-4-(prop-2-yn-1-yloxy)benzaldehyde (**1**) and 4-azido-1,2-dichlorobenzene (**2**) with the addition of CuAc as a catalyst and NaAsc as a reducing agent using dichloromethane/water (1:1) as a solvent system (Scheme 1).

In the IR spectra of the compound, as shown in Figure S5, the absence of an azide peak belongs to 4-azido-1,2-dichlorobenzene at 2120 cm^–1^ and the presence of the peak at 3155 cm^–1^ belongs to the target compound, showing the formation of the 1,2,3-triazole ring.^25^ In the IR spectra of compound **3**, the ν(C=O) stretching vibrations of benzaldehyde carbonyl were determined at 1644 cm^–1^ and ν(C–O) is identified at 1218 cm^–1^.^26^

**Figure 1:**
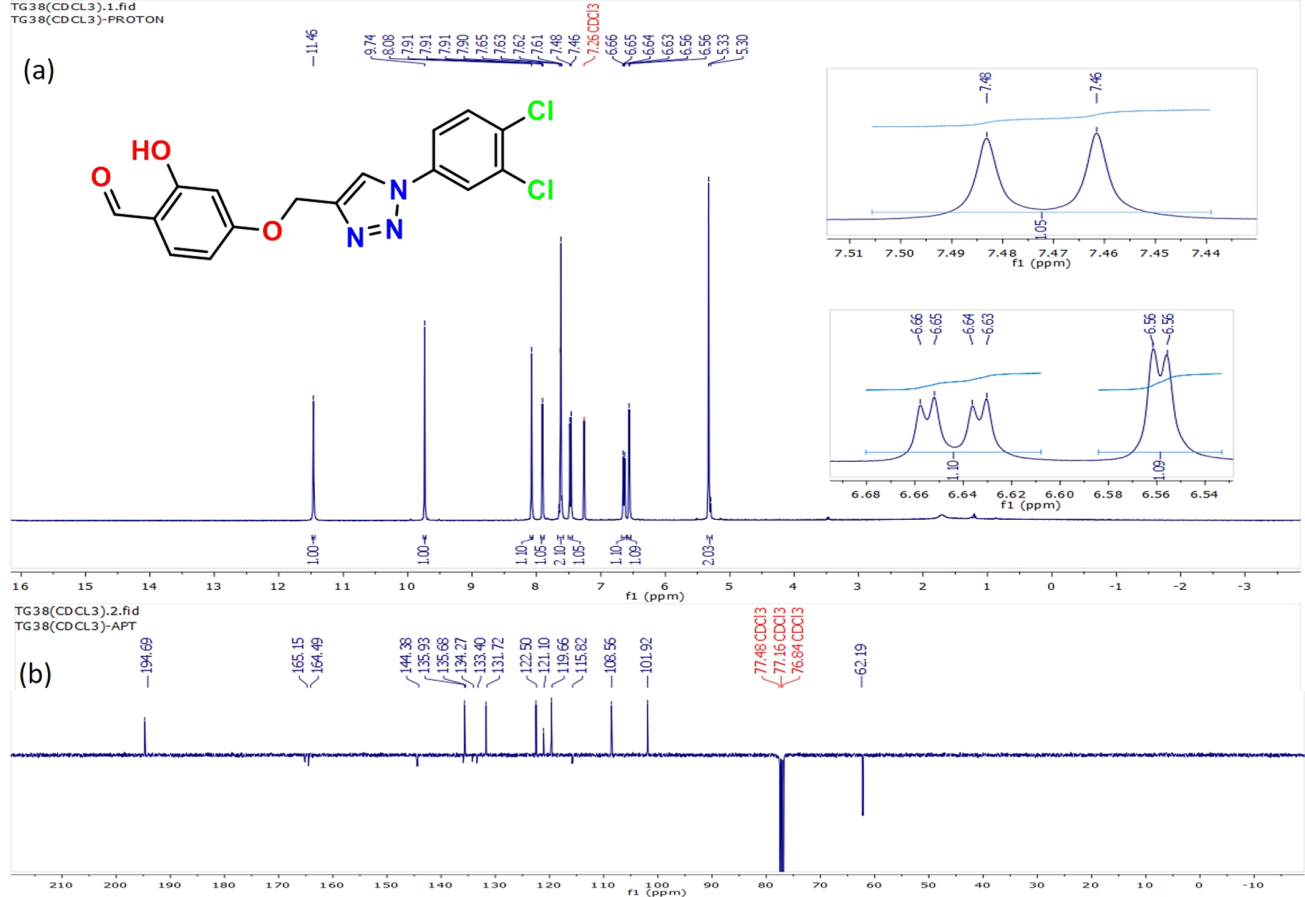
(a) ^1^H NMR spectra and (b) ^13^C APT NMR spectra of compound **3**.

The ^1^H NMR spectrum of 4-((1-(3,4-dichlorophenyl)-1*H*-1,2,3-triazol-4-yl)methoxy)-2-hydroxybenzaldehyde showed a singlet peak at δ 8.08 ppm due to the −CH of the 1,2,3-triazole ring, which confirms the formation of the 1,2,3-triazole moiety (Figure 1). Singlet signals at δ values of 11.46, 9.74, and 5.33 ppm correspond to the protons for (−OH), (−CHO), and (−CH_2_−), respectively. The aromatic protons appear as doublets at the δ values of 6.56, 6.64, 7.47, 7.62, and 7.91 ppm.^25,27,28^

The ^13^C NMR was applied as attached proton test (APT), where the signals belong to the CH/CH_3_ yield positive and signals belong to the C/CH_2_ yield negative. ^13^C NMR spectra of the compound showed peaks at δ values of 119.66 and 144.38 ppm, which were attributed to the C-5 and C-4 carbon atoms of the 1,2,3-triazole ring, respectively. The observed peak at 62.19 ppm was assigned to the methylene carbon attached to the C-4 position of the triazole ring. The signals belonging to the aldehyde carbonyl carbon atom appeared downfield at a δ value of 194.69 ppm. The signals at δ values of 165.15, 164.49, 135.93, 135.68, 134.27, 133.40, 131.72, 122.50, 121.10, 115.82, 108.56, and 101.92 ppm were assigned to the carbon atoms of aromatic rings. ^13^C NMR signal directions were in good agreement with the type of carbon atoms and had a good correlation with ^1^H NMR spectra when considering the attached protons to each carbon atom.

Spectral characterization data were similar to those previously reported for 1,2,3-triazole derivatives.^29−31^ The molecular formula was confirmed by ^1^H NMR, ^13^C APT NMR, FTIR, UV–vis, and elemental analysis.

### 2.2 Single-Crystal X-ray Structure

The spectroscopic data-based structure assignment of **3** was confirmed using single-crystal X-ray structural analysis. Table 1 contains the experimental details.

**Table 1:** Experimental Details for Compound **3**

CCDC	2240671
chemical formula	C_16_H_11_Cl_2_N_3_O_3_
*M*_r_	364.18
crystal system, space group	monoclinic, *P*2_1_/*c*
temperature (K)	273
*a*, *b*, *c* (Å)	18.0572 (6), 7.3509 (3), 12.1743 (4)
β (deg)	106.229 (4)
*V* (Å^3^)	1551.58 (10)
*Z*	4
radiation type	Mo Kα
μ (mm^–1^)	0.44
crystal size (mm)	0.12 × 0.10 × 0.05
Data Collection	
diffractometer	Bruker *APEX* II QUAZAR three-circle diffractometer
absorption correction	
no. of measured, independent, and observed [*I* > 2σ(*I*)] reflections	43989, 3832, 2645
*R*_int_	0.053
(sin θ/λ)_max_ (Å^–1^)	0.667
Refinement	
*R*[*F*^2^ > 2σ(*F*^2^)], w**R**(*F*^2^), *S*	0.053, 0.158, 1.04
no. of reflections	3832
no. of parameters	225
H-atom treatment	H atoms treated by a mixture of independent and constrained refinement
Δρ_max_, Δρ_min_ (e Å^–3^)	0.96, −0.47

**Figure 2:**
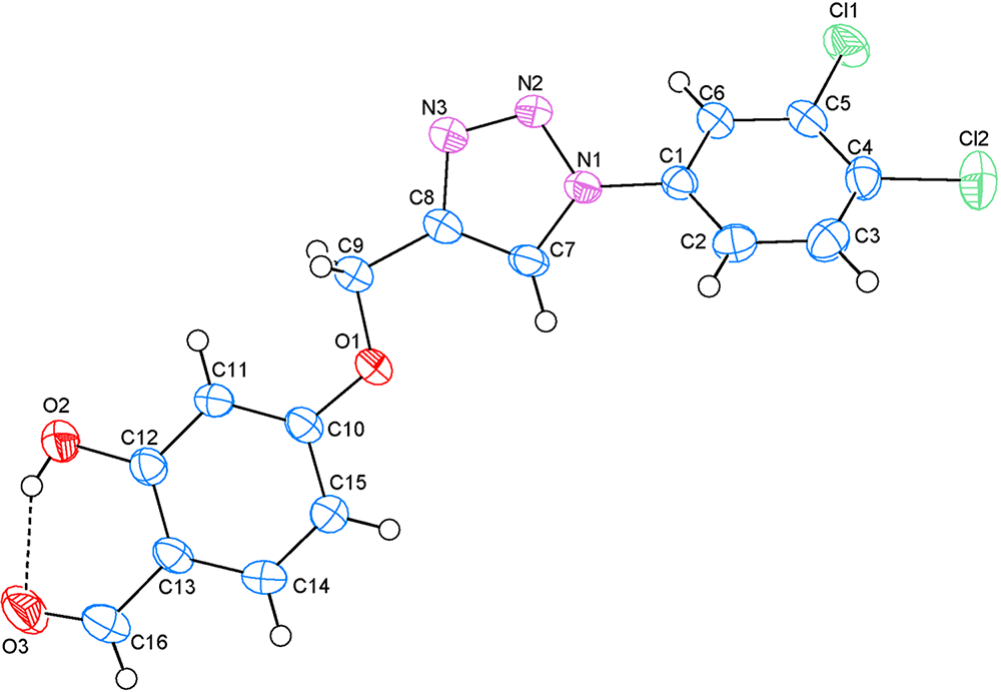
Asymmetric unit of compound **3** with the atom numbering scheme. Thermal ellipsoids are drawn at the 50% probability level. Dashed lines show the intramolecular O–H···O hydrogen bond.

The asymmetric unit along with the atom numbering scheme is depicted in Figure 2. Atoms Cl1, Cl2, C9, O1, O2, O3, and C16 are 0.0243 (8) Å, 0.0950 (9) Å, −0.0087 (28) Å, −0.0084 (20) Å, 0.0325 (24) Å, −0.0569 (24) Å, and −0.0204 (20) Å away from the best least-squares planes of adjacent rings *A* (C1–C6), *B* (N1–N3/C7/C8), and *C* (C10–C15), respectively. So, they are almost coplanar with adjacent rings.

The planar rings are oriented at dihedral angles of *A*/*B* = 35.76(8)°, *A*/*C* = 47.00(7)°, and *B*/*C* = 12.37(8)°. There is an intramolecular O–H···O hydrogen bond (Table 2).

**Table 2:** Hydrogen-Bond Geometry (Å, °) for Compound **3**^a^

*D*–H···*A*	*D*–H	H···*A*	*D*···*A*	*D*–H···*A*
O2–H2*A***···**O3	0.76 (4)	1.96 (3)	2.637 (3)	149 (4)
C2–H2**···**N2^i^	0.93	2.62	3.269 (3)	128
C6–H6**···**N3^ii^	0.93	2.60	3.509 (3)	167
C14–H14**···**O2^iii^	0.93	2.52	3.434 (3)	168

a Symmetry codes: (i) *x*, −*y* + 1/2, *z* – 1/2; (ii) −*x* + 1, −*y* + 1, −*z* + 2; (iii) *x*, −*y* + 3/2, *z* – 1/2.

**Figure 3:**
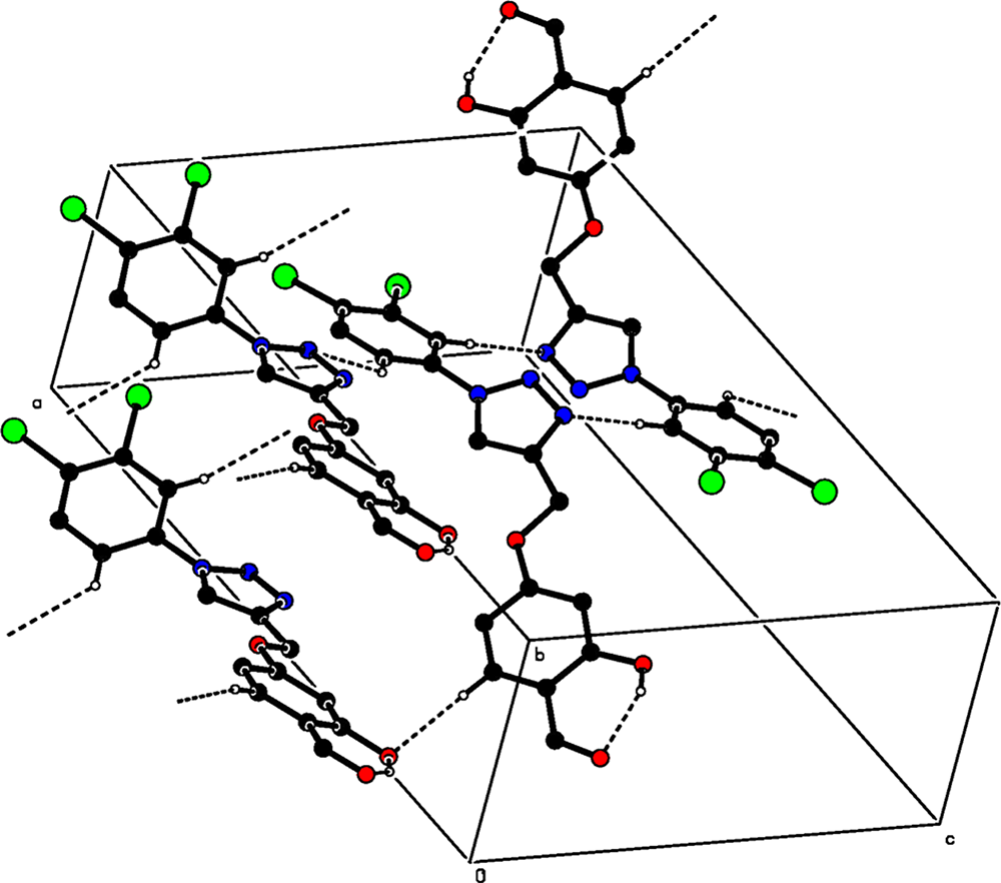
Partial packing diagram of compound **3**. Dashed lines show the intramolecular O–H**···**O and intermolecular C–H**···**O and C–H**···**N hydrogen bonds. For clarity, nonbonding hydrogen atoms have been omitted.

In the crystal structure of **3**, C–H**···**N and C–H**···**O hydrogen bonds link the molecules into a network structure (Table 2 and Figure 3), enclosing R_2_^2^(12) ring motifs.^32^ Further, π–π interactions between planar, *C* (C10–C15) rings, and [Cg3···Cg3*^i^*] of neighboring molecules help to consolidate the crystal packing [centroid–centroid distance = 3.8673 (13) Å; symmetry code: (i) −*x*, 2 – *y*, −*z*; Cg3 is the centroid of ring *C* (C10–C15)].

### 2.3 Hirshfeld Surface (HS) Analysis

**Figure 4:**
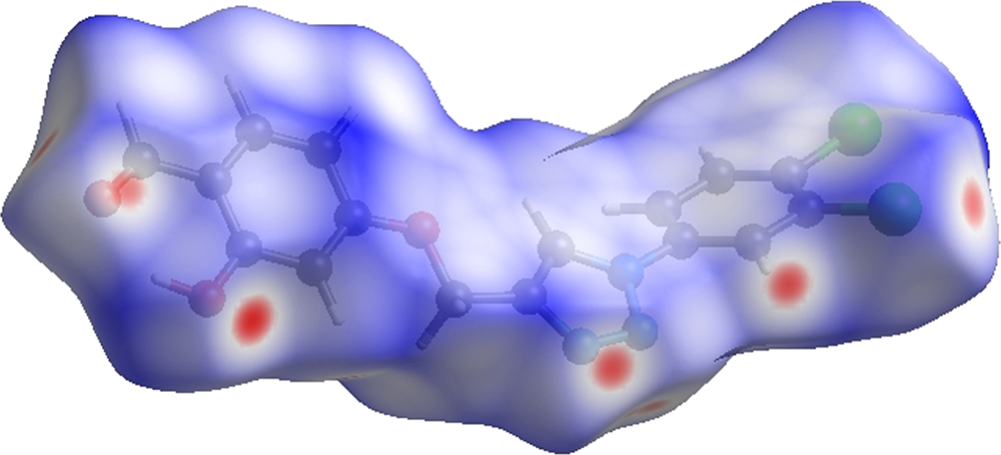
Three-dimensional (3D) Hirshfeld surface of compound **3** plotted over *d*_norm_ in the range of −0.1757–1.2160 au.

Hirshfeld surface (HS) analysis was carried out in order to visualize the intermolecular interactions in the crystal of **3**(33,34) by using Crystal Explorer 17.5.^35^ The *d*_norm_, electrostatic potential, and shape index are three different properties that can be used to map Hirshfeld surfaces. These are helpful in gathering additional information on weak intermolecular interactions. The white surface in the HS plotted over *d*_norm_ (Figure 4) denotes contacts with distances equal to the sum of van der Waals radii, while the red and blue colors refer to distances shorter (in close contact) or longer (distinct contact) than the van der Waals radii, respectively.^36^

**Figure 5:**
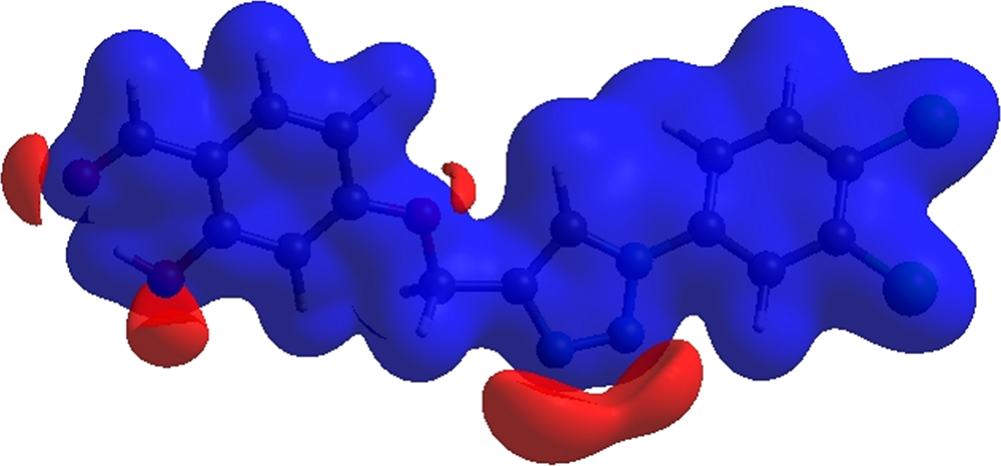
3D Hirshfeld surface of compound **3** plotted over electrostatic potential energy in the range of −0.0500–0.0500 au using the STO-3 G basis set at the Hartree–Fock level of theory.

In addition to appearing as blue and red regions corresponding to positive and negative potentials, respectively, on the HS mapped over electrostatic potential^37,38^ as shown in Figure 5, the bright red spots that appear show their roles as the respective donors and/or acceptors.

**Figure 6:**
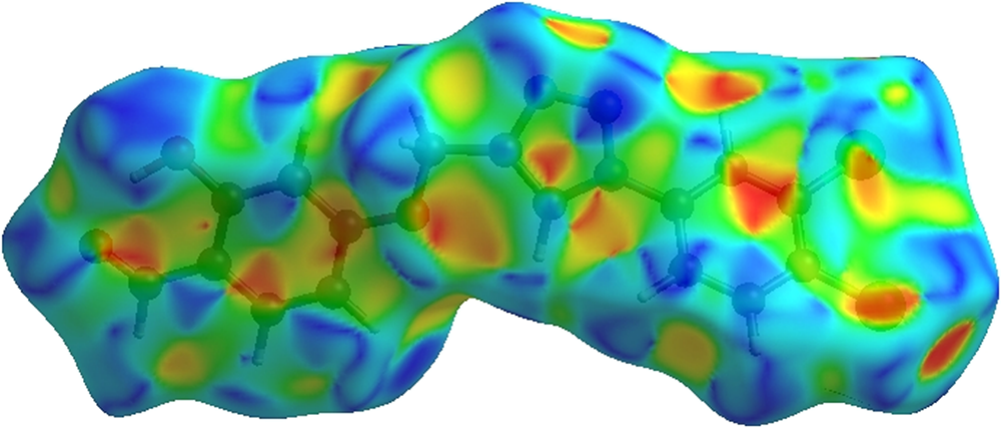
Hirshfeld surface of compound **3** plotted over shape index.

Hydrogen-bond donors are represented by blue regions, which have positive electrostatic potential, while hydrogen-bond acceptors are represented by red regions. The shape index of the HS is a tool to visualize the π···π stacking by the presence of adjacent red and blue triangles; if there are no adjacent red and/or blue triangles, no π···π interactions are present. Figure 6 clearly suggests that there are π···π interactions in **3**.

**Figure 7:**
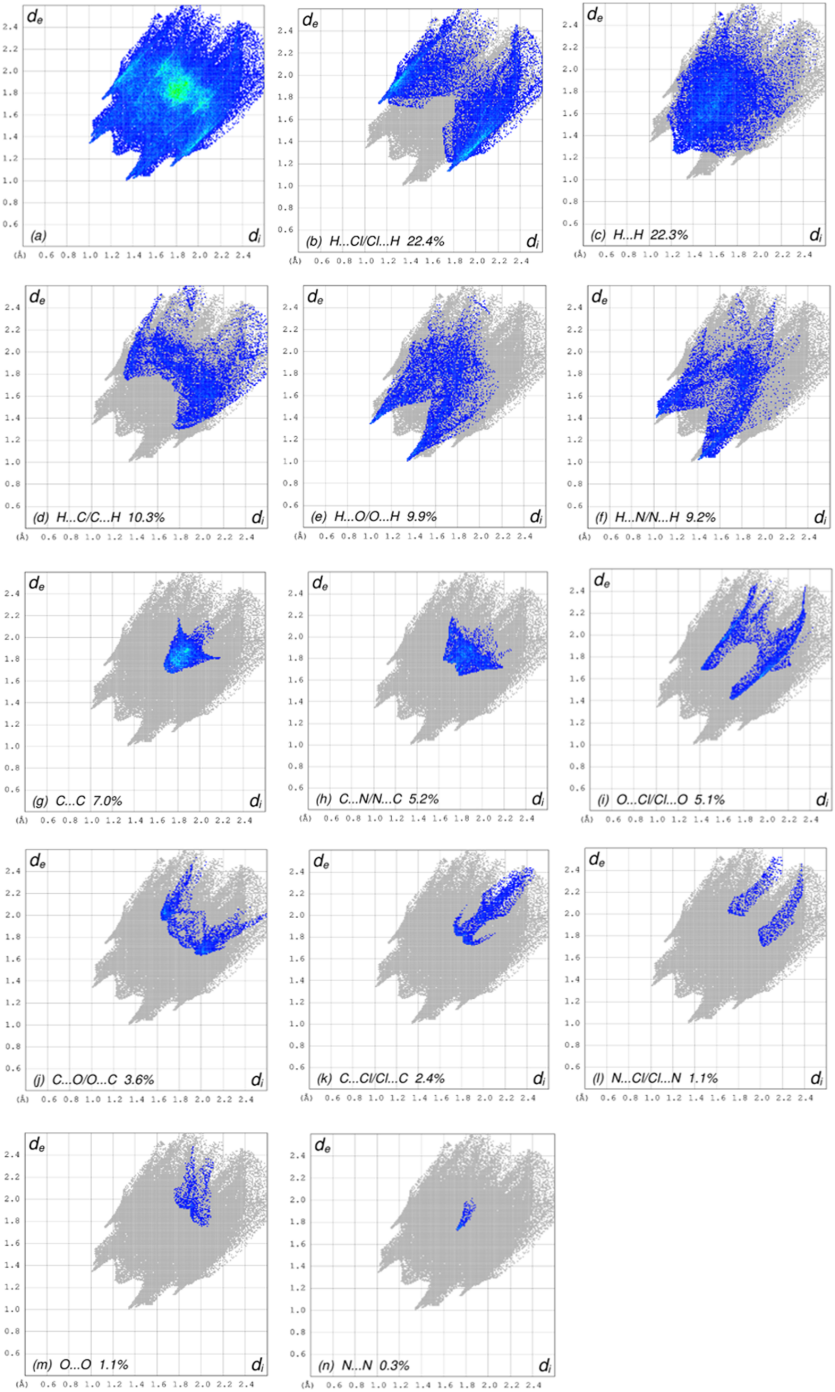
Full 2D fingerprint plots for compound **3**, showing (a) all interactions and divided into (b) H.. Cl/Cl···H, (c) H···H, (d) H···C/C···H, (e) H···O/O···H, (f) H···N/N···H, (g) C···C, (h) C···N/N···C, (i) O···Cl/Cl···O, (j) C···O/O···C, (k) C···Cl/Cl···C, (l) N···Cl/Cl···N, (m) O···O, and (n) N···N interactions. The *d_i_* and *d*_e_ values are the closest internal and external distances (in Å) from given points on the HS contacts, respectively.

The complex information contained in the crystal is summarized in two-dimensional (2D) fingerprint plots, which show the visual contents of the frequencies of the *d*_e_ and *d_i_* combinations across the surface of the molecule. The color of each point corresponding to the relative area of each *d*_e_ and *d_i_* combination is recognized as the contribution from different interatomic contacts. The uncolored region denotes no contribution to the HS, while the blue, green, and red correspond to the smallest, moderate, and largest contributions, respectively. The overall 2D fingerprint plot, Figure 7a, and those delineated into H···Cl/Cl···H, H···H, H···C/C···H, H···O/O···H, H···N/N···H, C···C, C···N/N···C, O···Cl/Cl···O, C···O/O···C, C···Cl/Cl···C, N···Cl/Cl···N, O···O, and N···N^39^ are illustrated in Figure 7b–n, respectively, together with their relative contributions to the Hirshfeld surface. The most important interaction is H···Cl/Cl···H contacts contribute 22.4% to the overall crystal packing, which is reflected in Figure 7b as the pair of spikes of the symmetrical distribution of points with the tips at *d*_e_ + *d_i_* = 2.86 Å. The H···H contacts are reflected in Figure 7c as widely scattered points of high density due to the large hydrogen content of the molecule with the tip at *d*_e_ = *d_i_* = 1.24 Å. In the absence of C–H···π interactions, the pair of characteristic wings in the fingerprint plot delineated into H···C/C···H contacts (Figure 7d, 10.3% contribution to the HS) has the tips at *d*_e_ + *d_i_* = 3.06 Å. The H···O/O···H (9.9%, Figure 7e) and H···N/N···H (9.2%, Figure 7f) contacts have symmetrical distributions of points with the pairs of spikes at *d*_e_ + *d_i_* = 2.34 Å and *d*_e_ + *d_i_* = 2.42 Å, respectively. The C···C (7.0%, Figure 7g) contacts have a bullet-shaped distribution of points with the tip at *d*_e_ = *d_i_* = 1.70 Å. The C···N/N···C (5.2%, Figure 7h) and O···Cl/Cl···O (5.1%, Figure 7i) contacts are observed with the tips at *d*_e_ + *d_i_* = 3.42 Å and *d*_e_ + *d_i_* = 3.12 Å, respectively. Finally, the C···O/O···C (3.6%, Figure 7j), C···Cl/Cl···C (2.4%, Figure 7k), N···Cl/Cl···N (1.1%, Figure 7l), O···O (1.1%, Figure 7m), and N···N (0.3%, Figure 7n) contacts have scattered points of low densities.

**Figure 8:**
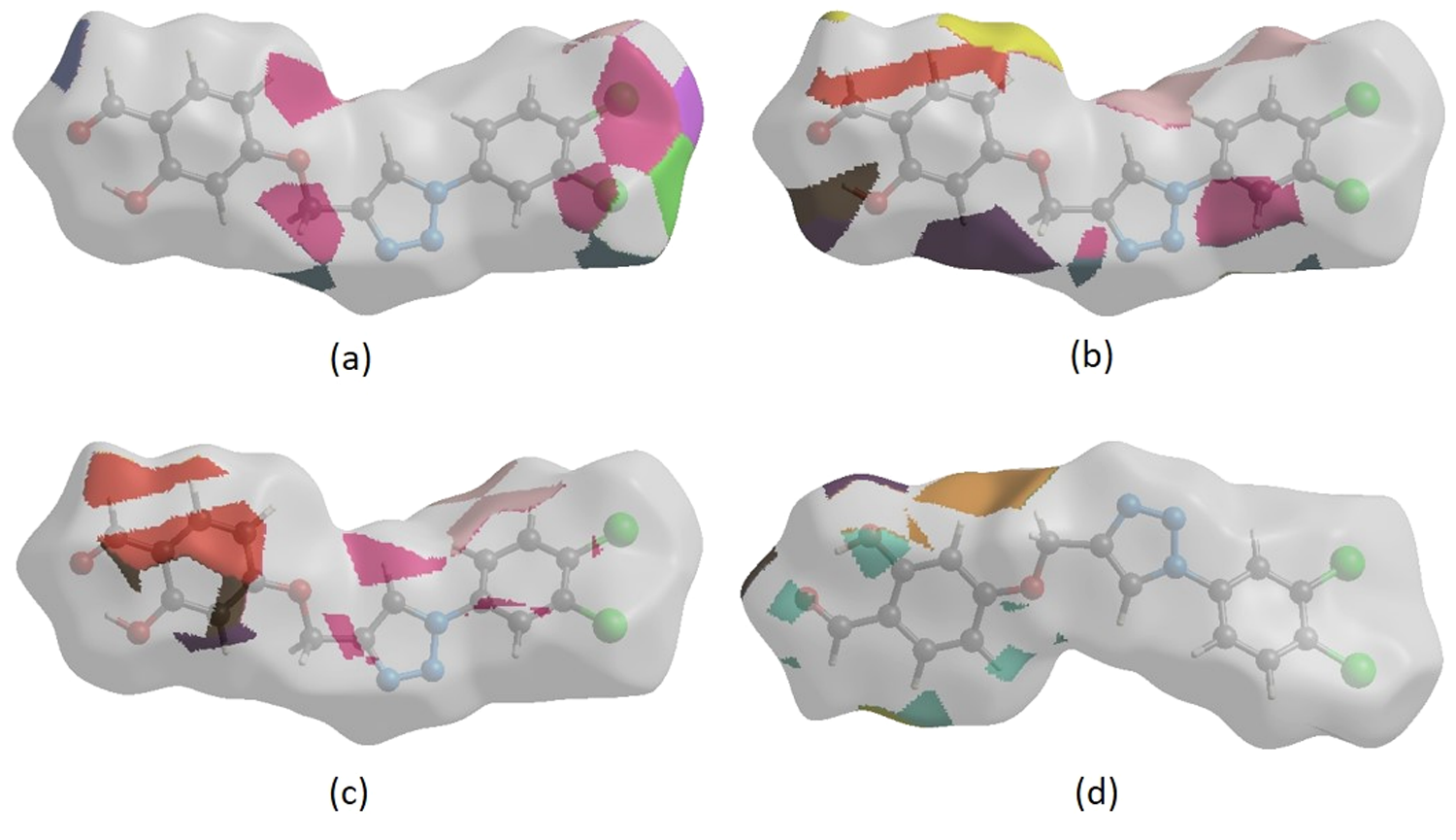
HS representations with the function *d*_norm_ plotted onto the surface for (a) H···Cl/Cl···H, (b) H···H, (c) H···C/C···H, and (d) H···O/O···H interactions.

The nearest neighbor coordination environment of a molecule can be determined from the color patches on the HS based on how close to other molecules they are. The HS representations with the function *d*_norm_ plotted onto the surface are shown for the H···Cl/Cl···H, H···H, H···C/C···H, and H···O/O···H interactions in Figure 8a–d, respectively.

The HS analysis confirms the significance of H-atom contacts in packing formation. The abundance of H···Cl/Cl···H, H···H, H···C/C···H, and H···O/O···H interactions suggests that van der Waals interactions and hydrogen bonding play major roles in the crystal packing.^40^

### 2.4 Crystal Voids

**Figure 9:**
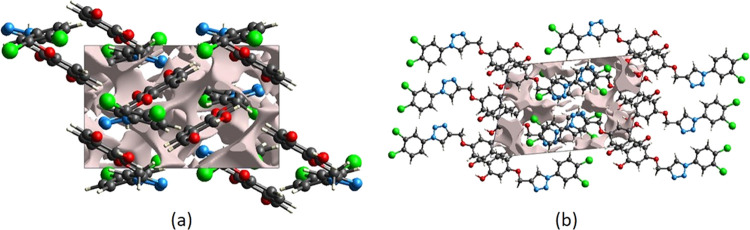
Views of voids in the crystal packing of compound **3**. Along the (a) *a*-axis and (b) *b*-axis.

The strength of the crystal packing determines how the crystal packing responds to the applied mechanical force. If there are significant empty spaces within the crystal packing, the molecules are not tightly packed and the crystal is easily broken by a small amount of applied external mechanical force. By adding the electron densities of the spherically symmetric atoms contained in the asymmetric unit, void analysis was carried out to assess the mechanical stability of the crystal packing.^41^ The void surface is calculated for the entire unit cell and defined as an isosurface of the procrystal electron density, where the void surface meets the boundary, and the capping faces are generated to form an enclosed volume. The volume of the crystal voids (Figure 9a,b) and percentage of free spaces in the unit cell are calculated to be 152.10 Å^3^ and 9.80%, respectively. As a result, there are no large cavities in the crystal packing.

### 2.5 Interaction Energy Calculations and Energy Frameworks

The intermolecular interaction energies are calculated with the CE–B3LYP/6-31G(d,p) energy model available in Crystal Explorer 17.5,^35^ and a cluster of molecules is generated by applying crystallographic symmetry operations to select the central molecule within a radius of 3.8 Å by default.^42^ The total intermolecular energy (*E*_tot_) is the sum of electrostatic (*E*_ele_), polarization (*E*_pol_), dispersion (*E*_dis_), and exchange–repulsion (*E*_rep_) energies^43^ with scale factors of 1.057, 0.740, 0.871, and 0.618, respectively.^44^ Hydrogen-bonding interaction energies (in kJ mol^–1^) are −18.7 (*E*_ele_), −4.2 (*E*_pol_), −24.2 (*E*_dis_), 23.7 (*E*_rep_), and −29.3 (*E*_tot_) for C2–H2**···**N2; −10.2 (*E*_ele_), −1.1 (*E*_pol_), −43.4 (*E*_dis_), 25.5 (*E*_rep_), and −33.6 (*E*_tot_) for C6–H6**···**N3; and −15.3 (*E*_ele_), −2.9 (*E*_pol_), −73.3 (*E*_dis_), 44.2 (*E*_rep_), and −54.8 (*E*_tot_) for C14–H14**···**O2.

**Figure 10:**
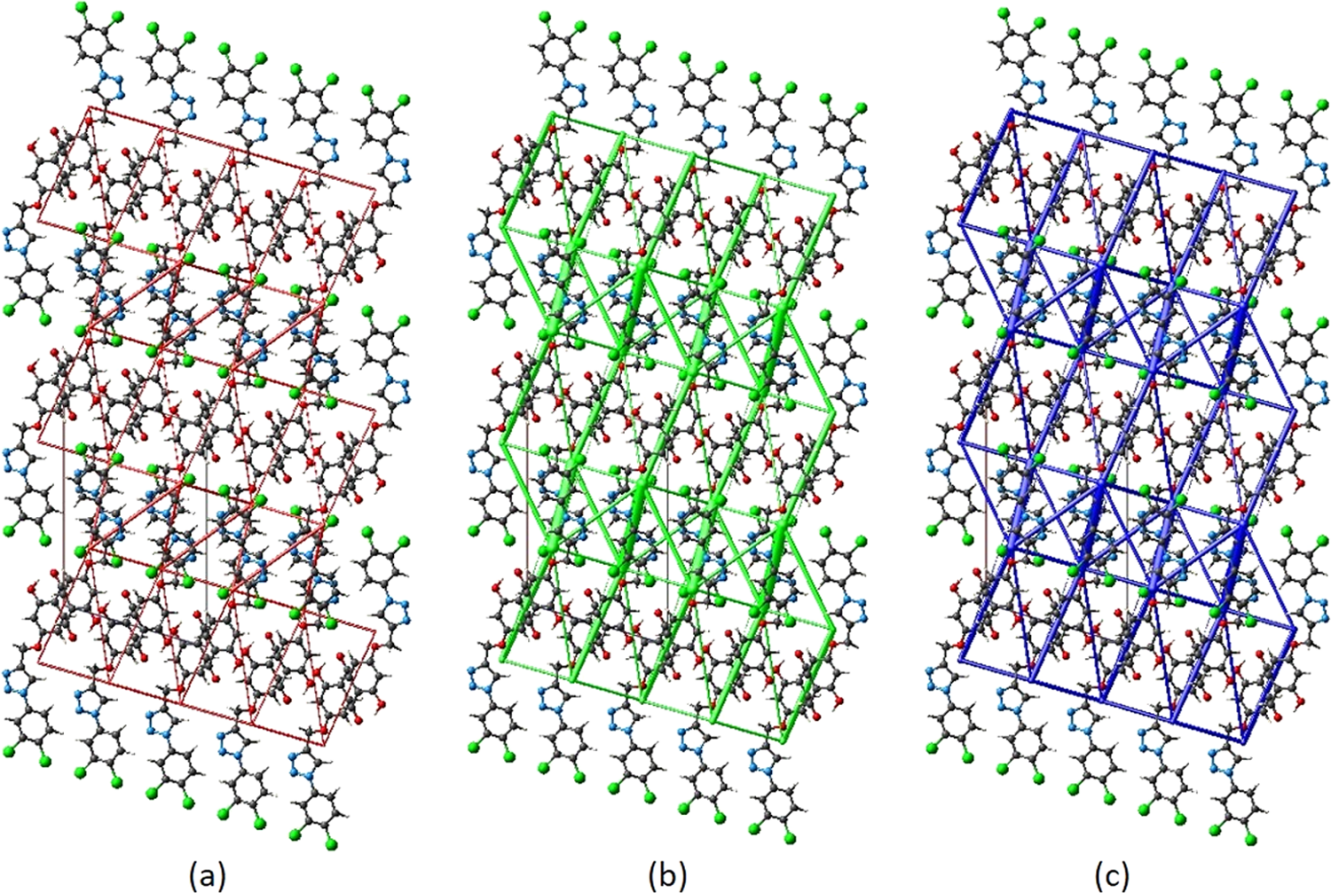
Energy frameworks for a cluster of compound **3** molecules viewed down the *b*-axis direction, demonstrating the (a) electrostatic energy, (b) dispersion energy, and (c) total energy diagrams.

The calculation of intermolecular interaction energies is combined with a graphical representation of their magnitude in energy frameworks.^43^ Energies between molecular pairs are represented by cylinders joining the centroids of two molecules, with the radius of the cylinder proportional to the relative strength of the corresponding interaction energies, which were scaled to the same factor of 80 with a cutoff value of 5 within 2 × 2 × 2 unit cells. Figure 10 shows energy frameworks for *E*_ele_ (red cylinders), *E*_dis_ (green cylinders), and *E*_tot_ (blue cylinders). The evaluation of the electrostatic, dispersion, and total energy frameworks shows that the dispersion energy contribution dominates the stabilization.

### 2.6 DNA Binding Studies

**Figure 11:**
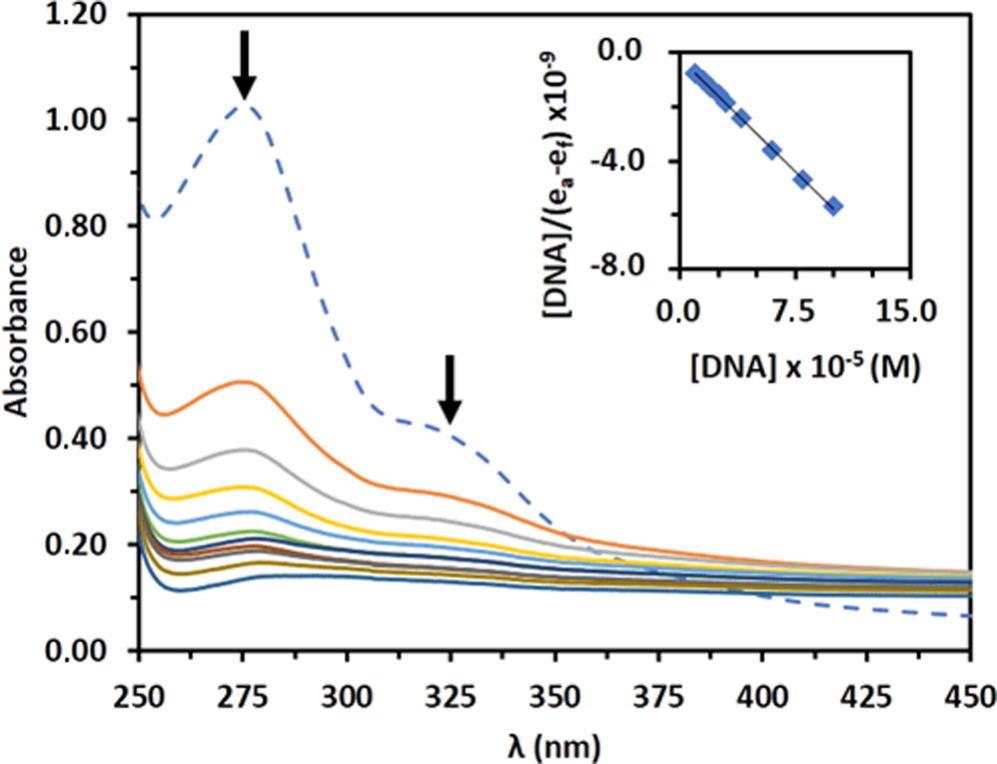
Absorption spectra of **3** (50 μM) in the absence (dashed line) and presence of increasing concentrations of CT-DNA. The intrinsic binding constant calculation plots for the spectral changes at 272 nm were given as the inset.

The use of absorption spectral titration to determine the interactions of potent anticancer agents with CT-DNA is an effective method.^45^ For this reason, the interactions between compound **3** and CT-DNA were investigated by measuring the absorption spectral changes when CT-DNA was added to a fixed concentration of the compound. Figure 11 shows the UV–vis spectra of compound **3** in the absence and presence of increasing concentrations (0–100 μM) of CT-DNA along with the inset of the Wolfe–Shimmer plot. Table 3 shows data on the UV–vis spectral changes and intrinsic binding constant (*K*_b_).

**Table 3:** CT-DNA Binding Data for Compound **3**

	λ_max_ (nm)						
compound	free	bound	Δλ (nm)	% H^a^	isosbestic point (nm)	*K*_b_ (M^–1^)^b^	Δ*G* (kJ·mol^–1^)
**3**	272	285	13.00	–86.18	380	3.00 × 10^5^	–31.25

a H, hypochromism.

b *y* = −6 × 10^–5^*x* – 2 × 10^–10^, *R*^2^ = 0.99.

The UV–vis spectra of **3** showed an absorption band at 272 nm, and this band revealed hypochromicity (86.18%) with increasing concentrations of CT-DNA. Upon the addition of CT-DNA, the absorption spectrum of compound **3** also showed a bathochromic shift (Δλ = 13 nm) within the bands at 272–285 nm, indicating an interaction between the electronic states of chromophores and DNA bases, possibly as a result of the intercalation. These observations are common for compound–DNA non-covalent interactions.^46^ Additionally, the isosbestic point at 370–390 nm indicates an equilibrium between the free and CT-DNA bound compound.^47^

The intrinsic binding constant (*K*_b_) was calculated based on titration data using the Wolfe–Shimmer equation.^48^*K*_b_ was determined by the slope-to-*y*-intercept ratio in the plot of [DNA]/(ε_a_ – ε_f_) versus [DNA], as detailed in the SI.

The *K*_b_ value for compound **3** was calculated as 3.00 × 10^5^ M^–1^, which is lower than those of typical intercalators such as EB with a *K*_b_ value of around 10^6^ M^–1^,^49^ but higher than the groove binders such as spermidine, methotrexate, and moxifloxacin, which have *K*_b_ values of 8.22 × 10^4^, 1 × 10^3^, and 9.4 × 10^4^ M^–1^, respectively.^50−52^ After considering the DNA binding abilities of compound **3** with previously reported 1,2,3-triazoles, we found that compound **3** had a similar *K*_b_ value around the order of 10^5^ M^–1^ as reported in the literature with a wide range of DNA binding properties and modes of interaction (e.g., intercalation or groove binding).^53−57^ In the case of intercalation, general spectral absorption changes for intercalation have been expected to be bathochromic (>15 nm) and hypochromic (>35%), whereas groove binding results in no bathochromism or around 6–8 nm shift.^58^ In light of these facts, our findings suggest that compound **3** was bound to CT-DNA via intercalation. Furthermore, Δ*G* for the DNA–compound **3** interaction was found to be −31.25 kJ·mol^–1^. A negative value revealed the spontaneity of the binding. However, further experiments were definitely needed to confirm the intercalation binding mode because of the lower bathochromic shift than typical intercalation interactions.

EB displacement studies were also employed using fluorescence spectroscopy. EB is a well-known DNA intercalator, intercalating with CT-DNA through its planar phenanthroline moiety to form an EB + CT-DNA complex. EB shows weak fluorescence intensity when excited around 520 nm. Intercalation of EB into DNA base pairs significantly increases the emission intensity of EB.^59^ If compound **3** or any compound competes with EB and intercalated between base pairs of EB-bound DNA, the emission will be decreased after the displacement of the EB molecules from the CT-DNA.

**Figure 12:**
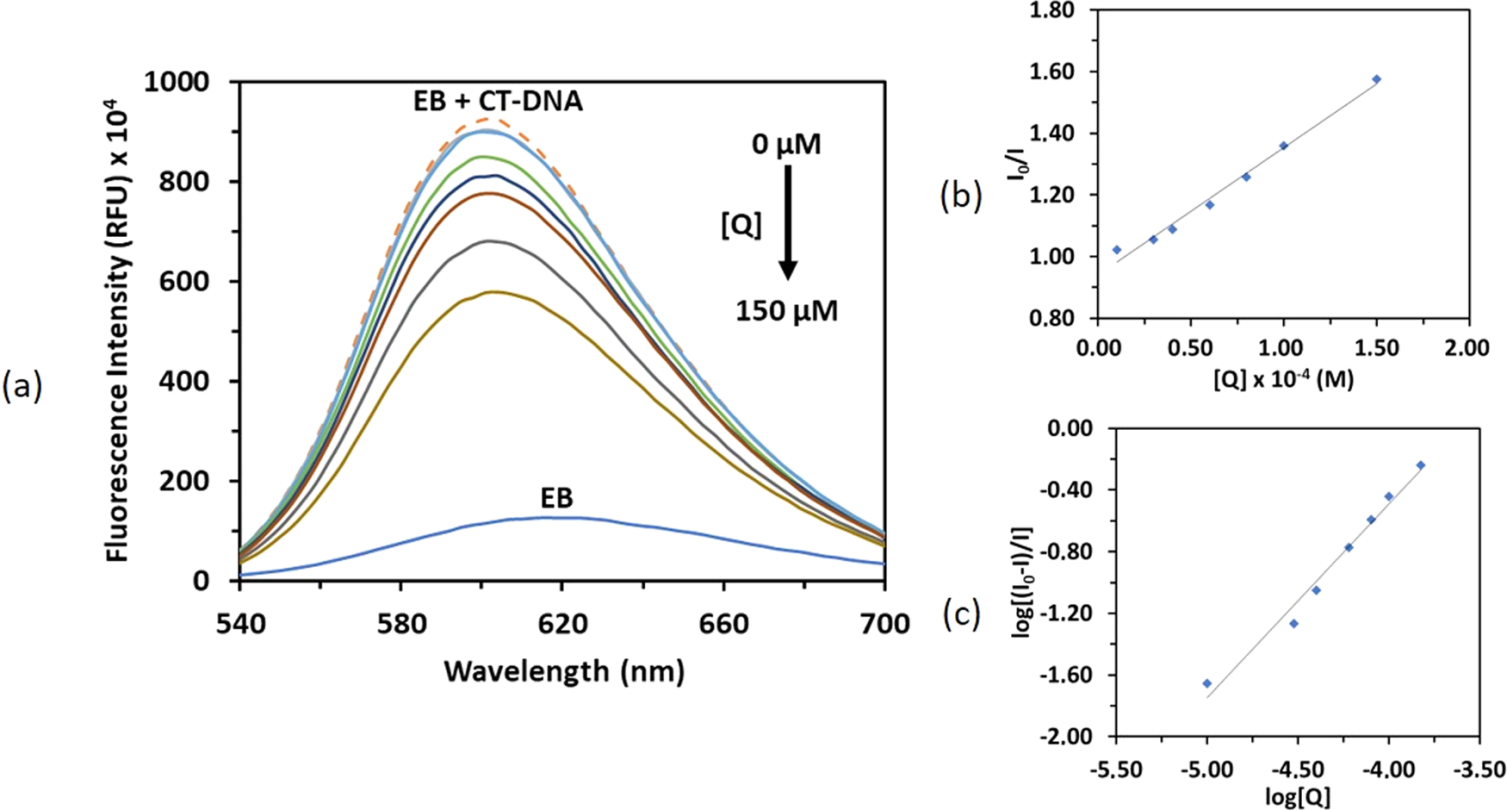
(a) Emission spectra of [EB (5 μM) + CT-DNA (25 μM)] in the presence of increasing concentrations of **3**. Arrow shows the decrease in emission intensity after additions of **3**. (b) Stern–Volmer plot of *I*_o_/*I* versus [*Q*] and (c) Scatchard plot of log[(*I*_o_ – *I*)/*I*] versus log [*Q*].

Increasing amounts of compound **3** (0–150 μM) were added into constant concentrations of [EB (5 μM) + CT-DNA (25 μM)] solution while the spectral changes in the fluorescence emission were monitored at 602 nm after excitation at λ_ex_ 510 nm. With the addition of increasing concentrations of compound **3** into the solution containing a fixed concentration of the EB + CT-DNA adduct, a decrease in the emission intensity of the 602 nm band is observed, as shown in Figure 12. This emission change in the EB + CT-DNA adduct after addition of compound **3** indicated that the studied compound displaced the EB from CT-DNA, which is a characteristic of the intercalative binding mode.^60^

The Stern–Volmer quenching constant (*K*_sv_) was calculated as 4.13 × 10^3^ M^–1^ from the slope of the plot of *I*_0_/*I* versus [*Q*] given in Figure 12b. The representative straight line plot for compound **3** according to the Stern–Volmer equation (*I*_o_/*I* = 1 + *K*_sv_[*Q*]) using emission spectral data is given in Figure 12b. The *K*_sv_ value suggests a moderate affinity of the compound to EB-bound CT-DNA and that it can competitively displace EB from DNA via an intercalative mode of binding (Table 4).

**Table 4:** Binding Data for the Interaction of Compound **3** with [EB + CT-DNA]

compound	*K*_sv_ (M^–1^)^a^	*K*_bin_ (M^–1^)^b^	*K*_q_ (M^–1^·s^–1^)	*n*
**3**	4.13 × 10^3^	3.45 × 10^4^	1.88 × 10^11^	1.26

a *y* = 4126.1*x* + 0.94, *R*^2^ = 0.99.

b *y* = 1.2569*x* + 4.5381, *R*^2^ = 0.98.

A typical linear Scatchard plot for compound **3** is shown in Figure 12c. Scatchard plots also provided the binding constant *K*_bin_, and the number of binding sites “*n*” values were calculated using the Scatchard equation log(*I*_o_ – *I*)/*I* = log *K*_bin_ + *n* log[*Q*]. The bimolecular quenching rate constant (*K*_q_) was calculated according to the equation *K*_sv_ = *K*_q_τ_0_ as 1.88 × 10^11^ M^–1^·s^–1^. This value is higher than those of typical dynamic quenchers (∼10^10^ M^–1^·s^–1^).^61−63^ According to the result, EB was displaced from CT-DNA statically instead of dynamically.^64^

The compound’s ability to displace EB and bind with CT-DNA was agreed with electronic absorption spectral data and indicated that the DNA binding mode of the compound was intercalation. Additionally, a molecular docking study was performed to gain insights into the mechanism and mode of DNA binding, and the docking results confirmed the experimental values pertaining to DNA binding studies.

### 2.7 BSA Binding Studies

Because of the structural similarity between bovine serum albumin and human serum albumin, drug interactions involving BSA are currently the subject of pharmaceutical studies.^65^ Fluorescence quenching and UV–vis absorption studies have been performed in order to understand the interaction mechanism between compound **3** and BSA.

**Figure 13:**
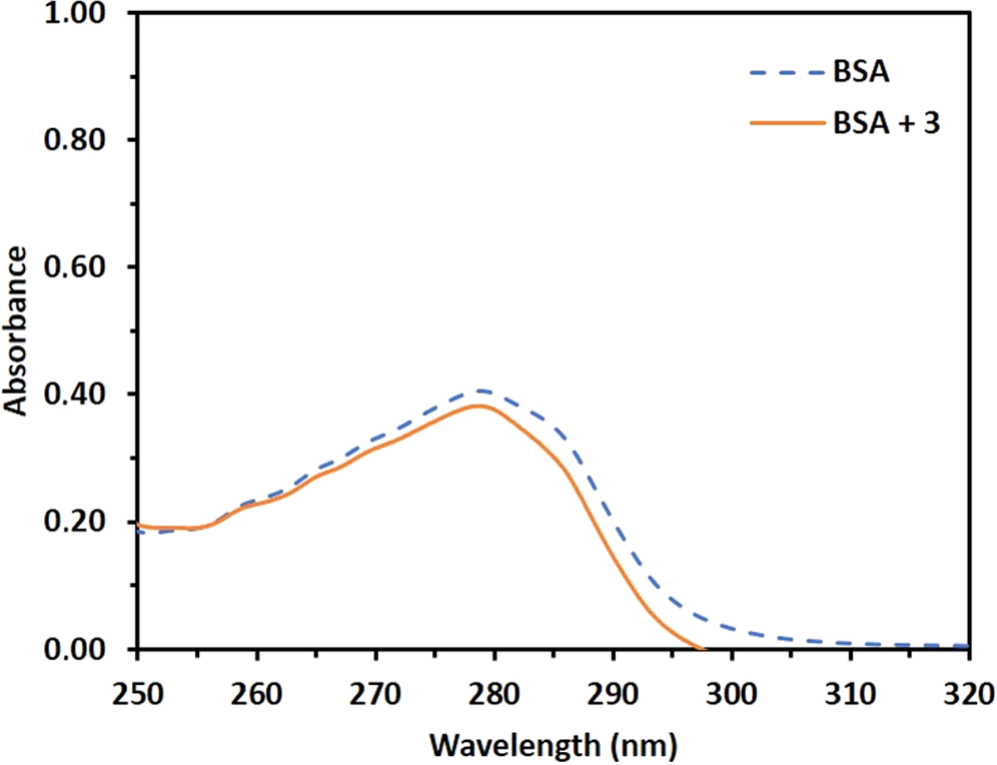
UV–vis spectra of BSA (10 μM) in the absence and presence of compound **3** (10 μM).

The UV–vis absorption spectrum of BSA provides a simple and straightforward way to investigate the type of quenching. A significant change in the absorption spectra of BSA is not expected in the case of a dynamic quenching mechanism; however, when a quencher forms a BSA adduct in its ground state for a static quenching mechanism, the UV–vis absorption spectra of BSA are expected to be changed.^66^ After addition of compound **3**, BSA absorption decreases with a slight blue shift (Δλ = ∼1 nm), indicating a possible static interaction between compound **3** and BSA (Figure 13).

Fluorescence spectroscopy is a well-known method for investigating the interaction mechanisms and binding affinities of potent compounds with bovine serum albumin. Tryptophan and tyrosine residues play a major role in the fluorescence properties of BSA. Tryptophan residues are primarily responsible for spectral changes related to protein conformational changes, denaturation, or substrate binding.^67,68^

**Figure 14:**
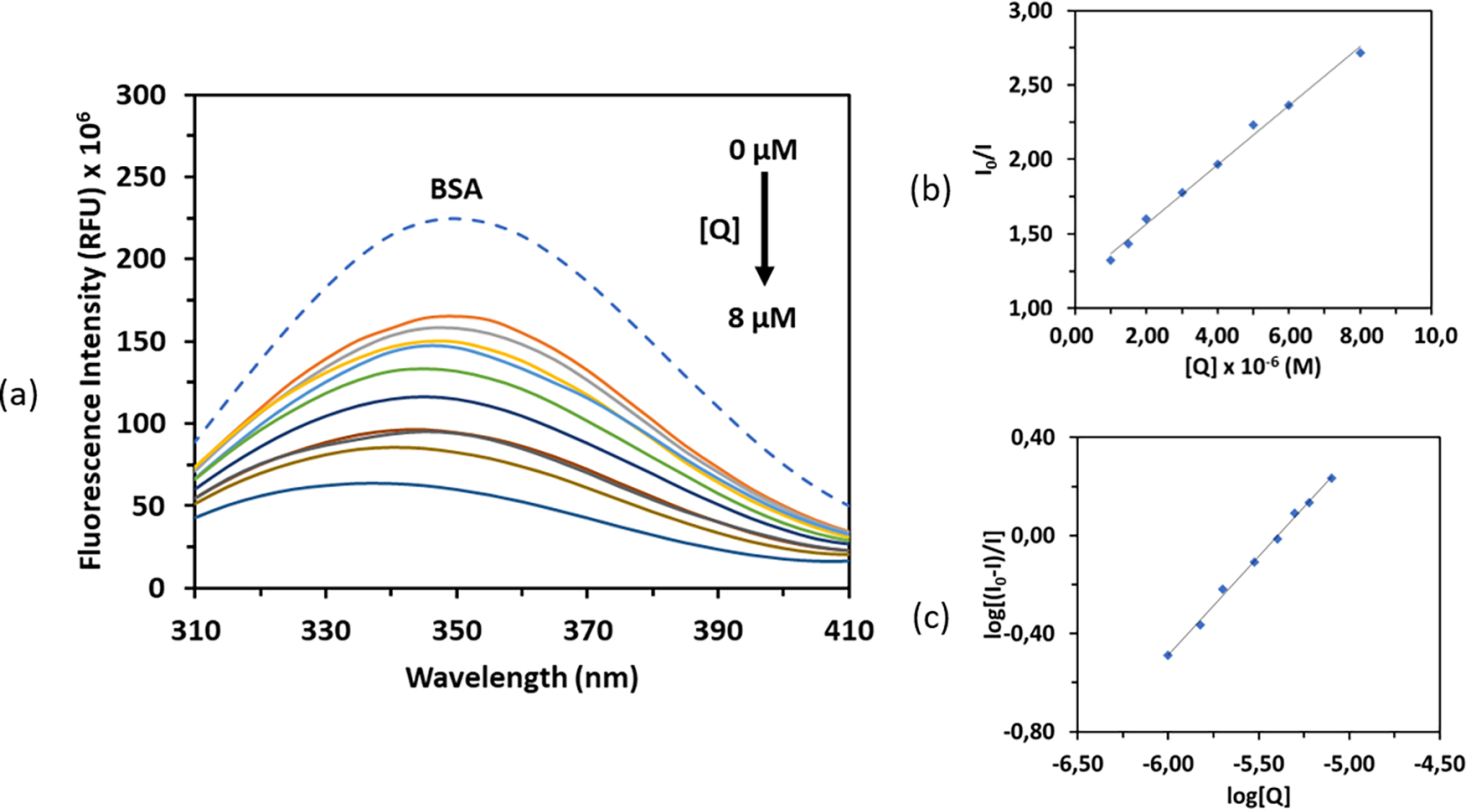
(a) Emission spectra of BSA (5 μM) in the presence of increasing concentrations of compound **3**. Arrow shows the decrease in the emission intensity after addition of **3**. (b) Stern–Volmer plot of *I*_o_/*I* versus [*Q*] and (c) Scatchard plot of log[(*I*_o_ – *I*)/*I*] versus log [*Q*].

Fluorescence spectroscopy experiments were conducted with 5 μM BSA and varying concentrations of compound **3** (ranging from 0 to 8 μM). The emission spectra were monitored in the 300–450 nm range after excitation at 280 nm. Figure 14 illustrates the fluorescence intensity of BSA at ∼345 nm, exhibiting a slight red shift (69.12%, 5 nm) as a result of quenching. The results showed that the fluorescence intensity consistently decreased as compound **3** was gradually added, indicating that compound **3** quenched the fluorescence of BSA as a result of interactions.

The quenching constant, *K*_sv_, has been determined using the Stern–Volmer equation and the *I*_0_/*I* versus [*Q*] plot (Figure 14b) in order to clarify the magnitude of the interaction and also the quenching type of compound **3** with bovine serum albumin. Furthermore, the Scatchard equation, log[(*I*_0_/*I*)/*I*] = log *K*_bin_ + *n* log [*Q*], was used to calculate the binding constant, where *K*_bin_ is the binding constant of **3** with BSA and *n* is the number of binding sites (Table 5). The values of *K*_bin_ and *n* were calculated from the log (*I*_0_/*I*)/*I* versus log [*Q*] plot, which is given in Figure 14c.

**Table 5:** Binding Data for the Compound **3**–BSA Interaction

compound	*K*_sv_ (M^–1^)^a^	*K*_bin_ (M^–1^)^b^	*K*_q_ (M^–1^·s^–1^)	*n*	Δ*G* (kJ·mol^–1^)
**3**	1.99 × 10^5^	2.18 × 10^4^	1.99 × 10^13^	0.80	–24.75

a *y* = 199382*x* + 1.1665, *R*^2^ = 0.99.

b *y* = 0.8044*x* + 4.3387, *R*^2^ = 0.99.

According to the Scatchard equation, the value of *n* supports the existence of a single binding site in BSA for compound **3**.^68^ The values of *K*_sv_ and *K*_bin_ for compound **3** further suggested that the compound interacts moderately with BSA. The bimolecular quenching constant, *k*_q_, was also computed using the equation *K*_sv_ = *k*_q_τ_0_, and the values of *k*_q_ and *n* are shown in Table 5. The *k*_q_ (∼10^13^ M^–1^·s^–1^) of compound **3** is higher than the maximum scatter collision quenching constant of diverse kinds of quenchers for biopolymer fluorescence (2 × 10^10^ M^–1^·s^–1^), indicating the existence of the static quenching mechanism.^69^ Furthermore, Δ*G* was calculated as −24.75 kJ·mol^–1^ and showed spontaneity of compound **3** binding with BSA.

### 2.8 Docking Studies on DNA, BSA, and Compound **3**

**Figure 15:**
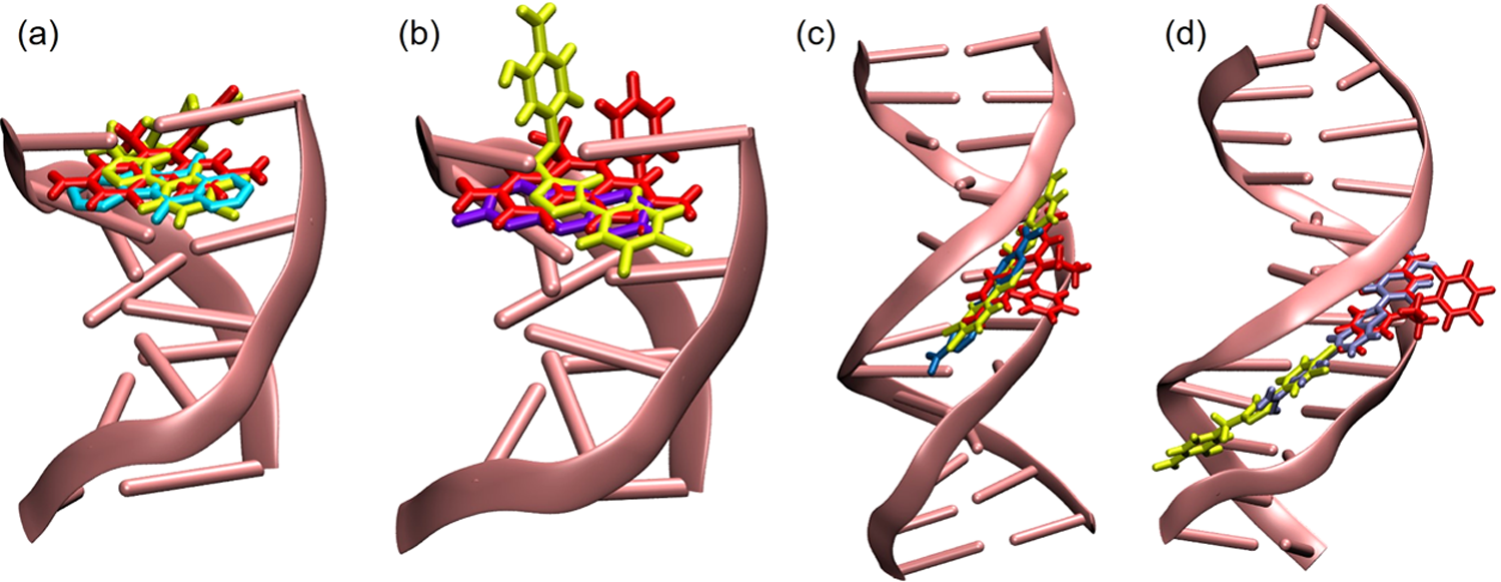
Binding poses obtained from docking studies performed with DNA and its respective crystal ligands with PDB IDs: (a) 1Z3F, (b) 3FT6, (c) 1D30, and (d) 8EC1. The DNA is shown in pink color. The ligands of 1ZF3, 3FT6, 1D30, and 8EC1 were shown in cyan, purple, crystal blue, and ice blue, respectively. Compound **3** and ethidium bromide were shown in yellow and red, respectively.

As explained in detail in the Materials and Methods section, we tested the performance of different docking schemes to identify the best method in terms of capturing experimentally obtained binding modes of the compounds in the crystal structures in complex with DNA. Accordingly, we demonstrated that the extra precision method, where enhanced sampling and enhanced planarity are implemented (named as XP3 here), gave smaller RMSD values between the docked and experimental poses (Table S1), so we performed the remaining docking studies using that method in this study. We used two differently shaped DNA molecules. In the first one, the intercalator distorted the DNA (Figure 15a,b), whereas in the second one, the molecule bound to the DNA from its groove (Figure 15c,d). We used ethidium bromide (Pubchem CID:14710), which is a well-known intercalating agent, as our control group in our docking studies.

We presented the binding poses pertaining to the ligand from the crystal complex, ethidium bromide, a compound that is known as an intercalator, and compound **3**, as shown in Figure 15. We demonstrated that compound **3** gave similar poses to those of ethidium bromide and the ligands present in the respective crystals. As explained in the methods, we performed docking including the whole DNA and observed that compound **3** preferred binding as an intercalator when partially open DNA was used (see Figure 15a,b), whereas it preferred binding to the minor groove when the DNA was intact (Figure 15c,d).

We also presented energy values obtained by these poses, as given in Table 6. In general, compound **3** gave closer binding energy values to those of ethidium bromide and the ligands when it bound to the partially open DNA, whereas the binding energy difference was bigger between compound **3** and both ethidium bromide and the crystal ligands in docking experiments performed in the groove of DNA, suggesting that compound **3** might act as an intercalating agent.

**Table 6:** Docking Energies Obtained by the XP3 Method

		PDB ID			
		1Z3F	3FT6	1D30	8EC1
GSCORE (kcal mol^–1^)	ligand (redock)	–6.41	–7.89	–11.86	–13.14
	ethidium bromide	–8.15	–7.39	–6.14	–6.13
	compound **3**	–5.19	–5.18	–2.91	–3.24

**Figure 16:**
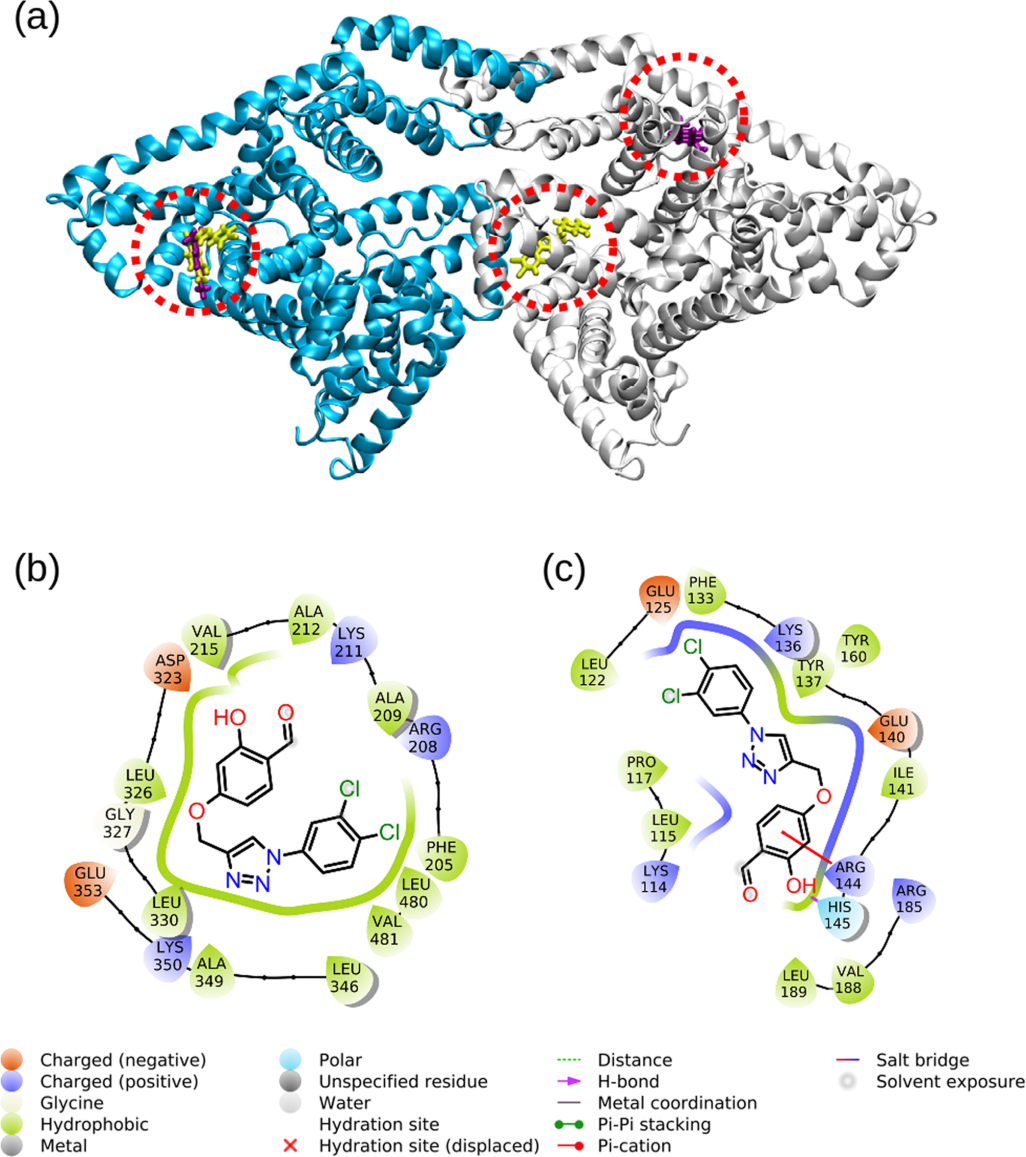
(a) Docking poses pertaining to naproxen (purple) and compound **3** (yellow) were shown with dashed circles on the crystal structure of BSA (PDB ID: 3V03). Chain A and chain B of BSA were shown in blue and white, respectively. Types of interactions formed between compound **3** and BSA in (b) chain A and (c) chain B are shown.

We also performed docking studies on compound **3** and BSA, which showed that compound **3** was found to be docked to the same region with naproxen in one of the chains of BSA, while they were docked to different regions on the other chain, as shown in Figure 16. The gscore value of compound **3** was found to be higher than that of naproxen (gscore_compound 3_ = −7 kcal·mol^–1^; gscore_naproxen_= −5 kcal·mol^–1^) in one of the chains, suggesting that compound **3** can bind and be carried by BSA. On the other hand, the binding energy of compound **3** was comparable to that of naproxen (gscore_compound 3_ = −6 kcal·mol^–1^) in the other chain of BSA. The interactions formed between naproxen and BSA in the crystal structure and docked models are shown in Figure S7.

### 2.9 Anticancer Activity

#### 2.9.1 MTT Assay

The MTT assay was used to determine whether compound **3** can inhibit cell proliferation in three cancer cell lines (MDA-MB-231, Caco-2, and LNCaP) and one normal cell line (HEK-293). Under identical conditions, the conventional anticancer drug etoposide was used as a positive control, and cisplatin was selected as parallel control.

**Figure 17:**
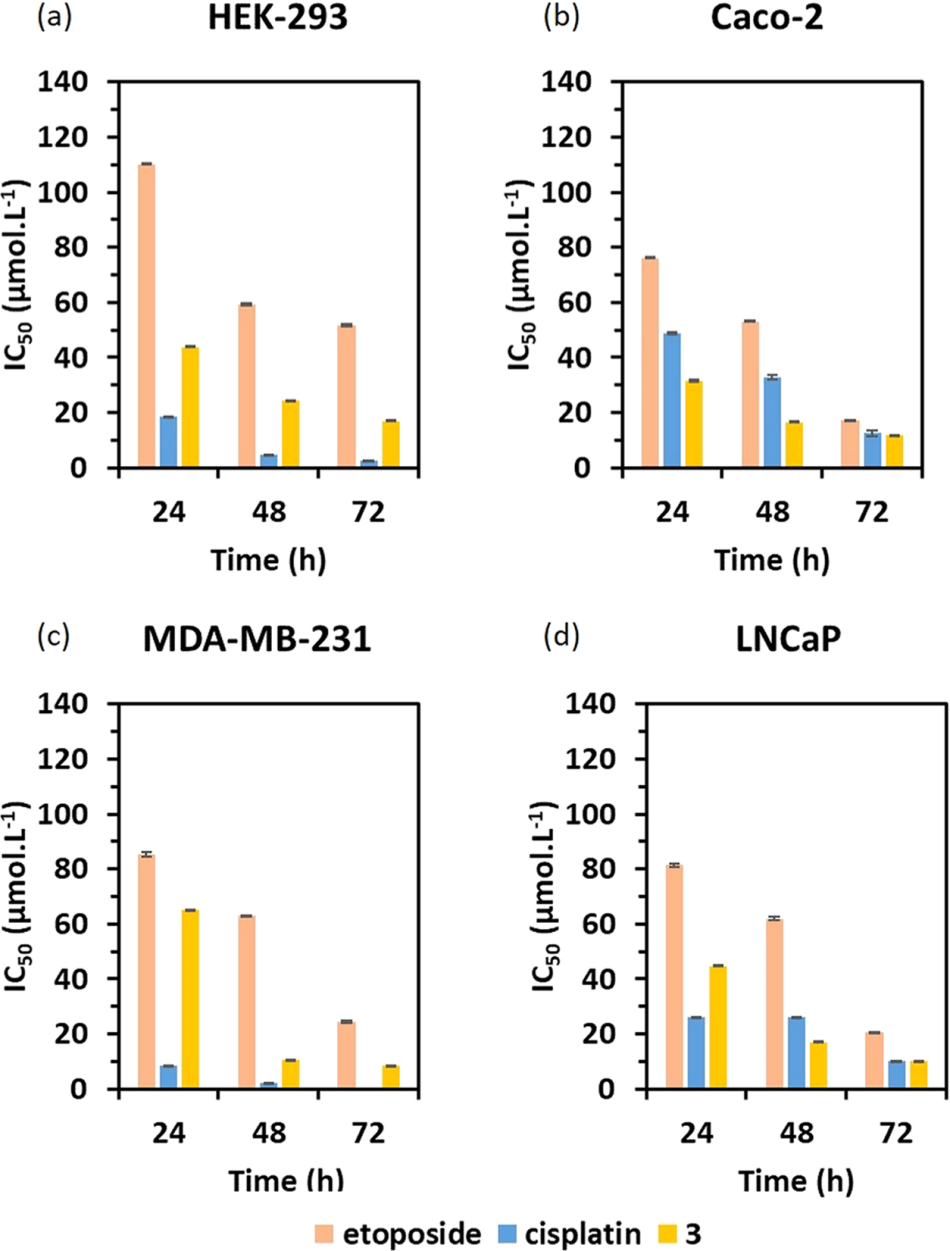
MTT Assay results of compound **3**, cisplatin, and etoposide in different cancer cell lines.

Compound **3** was found to have higher cytotoxic activity than etoposide for all cell lines in a dose-dependent manner after 24, 48, and 72 h. Compound **3** was found to be more cytotoxic than cisplatin in the Caco-2 and LNCaP (for 48 and 72 h) cell lines (Figure 17). The IC_50_ values of compound **3** (31.70 ± 0.34, 16.63 ± 0.27, 11.77 ± 0.01 μM) against Caco-2 cells for 24, 48, and 72 h were lower than those of cisplatin (48.92 ± 0.34, 32.69 ± 0.87, 12.5 ± 0.89 μM) and etoposide (76.27 ± 0.34, 53.81 ± 0.17, 17.52 ± 0.26 μM), respectively. For LNCaP cell lines, compound **3** showed better or equivalent inhibitory effects [IC_50_ values are 17.08 ± 0.26 (48 h) and 10.05 ± 0.03 (72 h)] than the cisplatin effects [IC_50_ values are 25.95 ± 0.48 (48 h) and 10.33 ± 0.28 (72 h)]. This suggests that compound **3** could inhibit cell growth in a dose- and time-dependent manner (Table 7). Compared to previously reported studies,^70−75^ cytotoxicity of compound **3** was effective as a potent cytotoxic agent.

**Table 7:** MTT Assay Results of Compound **3**, Cisplatin, and Etoposide

		IC_50_ (μM)			
time (h)	compound	HEK-293	MDA-MB-231	LNCaP	Caco-2
24	**3**	43.95 ± 0.20	65.03 ± 0.31	44.82 ± 0.09	31.70 ± 0.34
	cisplatin	18.62 ± 0.09	8.43 ± 0.01	26.05 ± 0.58	48.92± 0.34
	etoposide	110.29 ± 0.16	85.40 ± 0.74	81.47 ± 0.62	76.27 ± 0.34
48	**3**	24.44 ± 0.13	10.64 ± 0.03	17.08 ± 0.26	16.63 ± 0.27
	cisplatin	4.70 ± 0.01	2.19 ± 0.02	25.95 ± 0.48	32.69 ± 0.87
	etoposide	59.20 ± 0.32	63.17 ± 0.05	62.02 ± 0.61	53.81 ± 0.17
72	**3**	17.09 ± 0.08	8.39 ± 0.04	10.05 ± 0.03	11.77 ± 0.01
	cisplatin	2.43 ± 0.01	1.78 ± 0.01	10.33 ± 0.28	12.5 ± 0.89
	etoposide	51.62 ± 0.48	24.33 ± 0.44	20.79 ± 0.08	17.52 ± 0.26

Furthermore, the cytotoxicity of compound **3** and control drugs (cisplatin and etoposide) for HEK-293 suggests that compound **3**, cisplatin, and etoposide are all cytotoxic against HEK-293 cells, and compound **3** is less cytotoxic than the control cisplatin but more cytotoxic than the control etoposide. These results are noteworthy for calculating the selectivity index (SI) of the compounds studied against normal cells and cancer cells and predicting their therapeutic potential. The term selectivity index refers to how selectively the compounds can destroy cancer cells compared to normal cells. High SI values mean that cancer cells will be killed more quickly than normal ones.

SI values for compound **3**, etoposide, and cisplatin were calculated as SI(HEK/Caco-2) = 1.46, 1.10, 0.14, SI(HEK/MDA-MB-231) = 2.30, 0.94, 2.15, and SI(HEK/LNCap) = 1.43, 0.95, 0.18, respectively, showing that the cytotoxic selectivity of compound **3** was higher than those for both etoposide and cisplatin. Compared with the positive control of cisplatin, the SI of compound **3** was 10 times higher than that for Caco-2 cells and 8 times higher than that for LNCap cells (Table 8).

**Table 8:** SI Values (μM) of Compound **3** for 48 h^a^

compound	HEK-293/Caco-2	HEK-293/MDA-MB-231	HEK-293/LNCap
**3**	1.46	2.30	1.43
cisplatin	0.14	2.15	0.18
etoposide	1.10	0.94	0.95

a SI = IC_50_ on normal cells/IC_50_ on cancer cells.

The SI value of compound **3** for the MDA-MB-231 cancer cell line was found to be greater than 2, which is considered acceptable selectivity for SI definition. The findings indicate that the compound’s anticancer activity is valuable, as evidenced by its low cytotoxicity against healthy cells and moderate cytotoxicity against cancer cells. Consequently, compound **3** has potential for selective cytotoxicity against cancer cells.

#### 2.9.2 Apoptosis Assay

**Figure 18:**
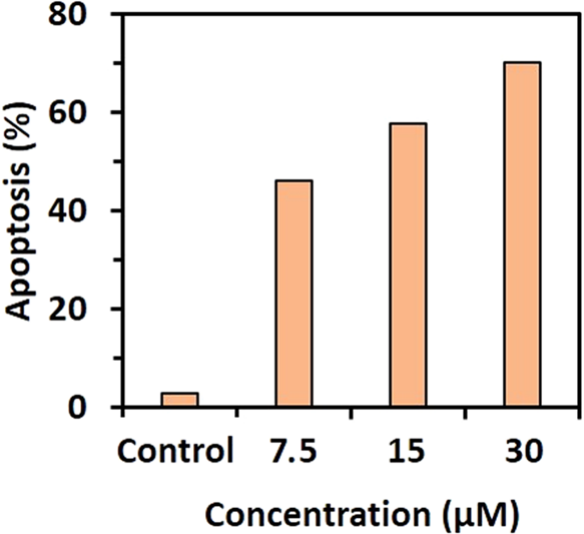
Bar diagram of percent apoptotic cells (early + late) according to Figure S8.

Following findings that compound **3** impacts the viability of cancer cell lines and displays the most promising antitumoral activities toward Caco-2 cells, we analyzed the Annexin V/propidium iodide (PI) staining in cell culture media by flow cytometry to investigate whether apoptosis accounted for the cytotoxicity of compound **3** on Caco-2 cells. The cells exposed to compound **3** (7.5, 15, and 30 μM) showed high levels of Annexin V and PI positive staining compared to the control (without compound **3**), suggesting the induction of cell death by apoptosis (Figure 18).

As shown in Figure 18, after treatment with compound **3** at different concentrations (0, 7.5, 15, and 30 μM) for 48 h, the percentages of apoptosis on Caco-2 cells were 2.8, 46.09, 57.77, and 70.25%, respectively. The Annexin V/PI result indicated that compound **3** could induce apoptosis of cells with concentration dependence.

#### 2.9.3 MMP and ROS Assay

**Figure 19:**
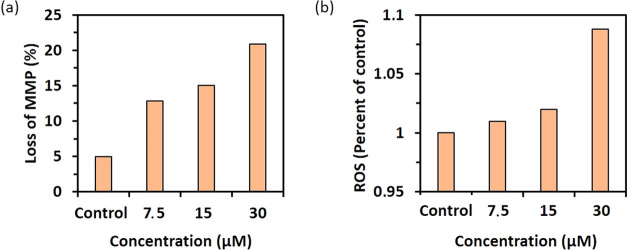
(a) Compound **3**-treated Caco-2 cells show a dose-dependent increase in MMP loss. (b) Flow cytometry analysis of ROS production in compound **3**-treated Caco-2 cells. Compound **3** caused a dose-dependent increase in ROS levels in cancer cells.

However, we decided to investigate other mechanisms besides cell death under the action of compound **3**, such as MMP and ROS. In many cells, morphological and molecular changes in mitochondria are crucial stages in apoptosis. By staining Caco-2 cells with the intrinsically fluorescent dye TMRE, we were able to detect a loss of mitochondrial membrane potential after 48 h of treatment with compound **3**. According to the results, compound **3** caused a significant increase in MMP loss in Caco-2 cells in a dose-dependent manner (Figure 19a).

The generation of ROS within cells is closely linked to the induction of apoptosis. Figure 19b depicts the production of ROS by compound **3** on Caco-2 cells for 48 h at 0, 7.5, 1.5, and 30 μM concentrations. The results showed that adding compound **3** increased the fluorescence intensity, indicating the generation of reactive oxygen species, proving that compound **3** could induce cell apoptosis via ROS generation.

## 3 Conclusions

In the current study, a new 1,4-disubstituted 1,2,3-triazole derivative (**3**) was synthesized using an azide and alkyne compound via click chemistry. In addition to various spectroscopic methods, structural confirmation of the compound was carried out by SCXRD, which revealed a monoclinic system with the *P*2_1_/*c* space group. HS analysis was also applied, and van der Waals interactions and hydrogen bonding were found to be the dominant types of interactions. The volume of crystal voids showed that there was no large cavity in the crystal packing. The investigation of the electrostatic, dispersion, and total energy frameworks indicates that stabilization is dominated by the dispersion energy contribution.

In vitro and in silico investigations on DNA/BSA binding activity of compound **3** showed that DNA interaction via intercalation mode (*K*_b_: 3.00 × 10^5^ M^–1^) and both polar and hydrophobic interactions via BSA and both bindings were spontaneous as we obtained negative Δ*G* values. In vitro MTT cytotoxic activities of the compound gave two times better cytotoxicity activity after 48 h in human colorectal adenocarcinoma cell line Caco-2 (16.63 ± 0.27 μM) than cisplatin (32.69 ± 0.87 μM) and nearly three times better than etoposide (53.81 ± 0.17 μM). Also, it had the least effect on human normal cell line HEK-293 compared to the control drug cisplatin. These findings reflect that the SI values of compound **3** were higher than those for both etoposide and cisplatin on all cancer cells. In addition, compound **3** induced apoptosis in the Caco-2 cancer cell line. The wet lab and computational investigations suggest that the 1,2,3-triazole derivative (**3**) may be utilized as an alternative cytotoxic agent for cancer therapy with no adverse effects on healthy cells. However, these results must be validated in vivo, and further experiments are needed.

## 4 Experimental Section

### 4.1 Materials and Methods

All reagents were purchased from Merck/Sigma. Working solutions were prepared using doubly distilled water. A LECO 932 CHNS analyzer was used for microanalysis (C, N, H), and a Bruker 400 MHz spectrometer was used to perform NMR analysis. IR spectra were recorded with a Nicolet iS10-ATR (Thermo-Scientific). DNA and BSA interaction studies were performed using a T80+ UV–vis spectrophotometer (PG) and a SpectraMax i3x Multi-Mode microplate reader (Molecular Devices). X-ray data collections were performed with a Bruker APEX II QUAZAR three-circle diffractometer using Mo Kα radiation (λ = 0.71073 Å). 2-Hydroxy-4-(prop-2-yn-1-yloxy)benzaldehyde (**1**) and 4-azido-1,2-dichlorobenzene (**2**) were synthesized according to previously reported methods,^76,77^ and their detailed syntheses and characterization data are given in the Supporting Information (SI).

### 4.2 Synthesis of 1,4-Disubstituted 1,2,3-Triazole

The compound was prepared according to a previously reported method by us with minor modifications.^25^ Briefly, Cu(CO_2_CH_3_)_2_·H_2_O (0.040 g, 0.20 mmol) and sodium ascorbate (0.079 g, 0.40 mmol) were added to a solution of 2-hydroxy-4-(prop-2-yn-1-yloxy)benzaldehyde (0.176 g, 1 mmol) and azide derivative (1.20 mmol) in 10 mL of a 1:1 mixture of H_2_O/CH_2_CI_2_. After stirring for 8 h at RT, the mixture was diluted with 10 mL of H_2_O/CH_2_CI_2_ (1:1), and the organic layer was separated, washed with water (1x), 1 M EDTA (3x), and brine (2x), dried over Na_2_SO_4_, and filtered. The solvent evaporated to give a brownish or yellowish residue. Then, the residue was dissolved in the minimum amount of CH_2_CI_2_, and an off-white product was precipitated by adding 50 mL of *n*-hexane. The pure compound was filtered and dried, and single crystals suitable for X-ray analysis were obtained by the recrystallization of the compound from a methanol/ethyl acetate mixture at room temperature (Scheme 1).

#### 4.2.1 4-((1-(3,4-Dichlorophenyl)-1*H*-1,2,3-triazol-4-yl)methoxy)-2-hydroxybenzaldehyde

Yield: 89%. mp: 144–145 °C. FTIR (ATR, ν, cm^–1^): 3155 (C–H)_tz_, 3074–2837 (Ar–H), 1218 (C–O), 1644 (C=O). ^1^H NMR (400 MHz, CDCI_3_) δ (ppm): 11.46 (s, 1H), 9.74 (s, 1H), 8.08 (s, 1H), 7.91 (dd, *J* = 1.9, 1.0 Hz, 1H), 7.62 (d, *J* = 1.8 Hz, 2H), 7.47 (d, *J* = 8.6 Hz, 1H), 6.64 (dd, *J* = 8.6, 2.4 Hz, 1H), 6.56 (d, *J* = 2.4 Hz, 1H), 5.33 (s, 2H). ^13^C APT NMR (101 MHz, CDCI_3_) δ (ppm): 194.69, 165.15, 164.49, 144.38, 135.93, 135.68, 134.27, 133.40, 131.72, 122.50, 121.10, 119.66, 115.82, 108.56, 101.92, 62.19. Anal. calcd. for C_16_H_11_Cl_2_N_3_O_3_: C, 52.77; H, 3.04; N, 11.54. Found C, 52.65; H, 3.05; N, 11.54.

### 4.3 X-ray Crystallography

The crystallographic data were processed by SHELX program packages [SHELXT 2018/2^78^ and SHELXL-2018/3^79^] to solve and refine the molecular structure. The structure visualizations were obtained using ORTEP-3^80^ and PLATON^81^ programs. Materials were prepared for publication using the WinGX^80^ publication routine. The hydroxy and methine hydrogen atoms are located in a difference Fourier map and refined freely. The positions of the C-bound hydrogen atom were calculated geometrically at distances of 0.93 Å (for aromatic CH), 0.97 Å (for CH_2_), and 0.96 Å (for CH_3_) and refined using a riding model with the constraints of U_iso_(H) = *k* X U_eq_ (C), where *k* = 1.5 for methyl H atoms and *k* = 1.2 for other H atoms. Crystallographic data for the structure described in this paper have been deposited with the Cambridge Crystallographic Data Centre as Supporting Information, CCDC No. 2240671.

### 4.4 DNA Binding Experiments

The DNA binding activity of the compound was investigated using UV–vis and fluorescence spectral studies in accordance with our previous studies.^59,82^

All titrations were performed in 10 mM Tris-HCl buffer with a pH of 7.4 at room temperature. The UV–vis absorption studies were performed by adding increasing amounts of DNA (0–100 μM) to a solution of a fixed concentration (50 μM) of the compound. The Wolfe–Shimmer equation^48^ was used to calculate the intrinsic binding constant (*K*_b_), and the van’t Hoff equation was used to calculate the Gibbs free energy (Δ*G*).

The competitive emission quenching experiment was carried out using a well-known intercalating agent and fluorescent probe for DNA, ethidium bromide (EtBr).^83^ The compound in varying concentrations (0–150) was added to the [EB (5 μM) + CT-DNA (25 μM)] adduct. The excitation wavelength was fixed to 510 nm, and the emission spectra were recorded (λ_em_ = 500–700 nm). The quenching efficiency of the compound was analyzed using the Stern–Volmer and Scatchard equations, and the Stern–Volmer (quenching) constant, *K*_sv_, the bimolecular quenching rate constant, *k*_q_, binding constant, *K*_bin_, and the number of binding sites per nucleotide, *n*, were calculated.^84^

The detailed experimental procedures including solution preparations and calculations of the binding data for both absorption studies and fluorescence studies are provided in the SI.

### 4.5 BSA Binding Experiments

The BSA–compound interactions are studied by comparing the UV–vis spectra (200–400 nm) of BSA (10 μM) alone and a mixture of [BSA (10 μM) + compound **3** (10 μM)] at room temperature in 10 mM Tris-HCl buffer with a pH of 7.4.^85^

BSA fluorescence spectral studies of the compound and related calculations were the same as those performed for the [CT-DNA + EB] quenching method described in Section 4.5. Detailed experimental procedures and the calculations of the binding data are given in the SI.

### 4.6 Docking Studies on DNA, BSA, and Compound **3**

As we opt to examine the binding mode of compound **3** (intercalation vs groove binding), we first investigated the performance of different docking schemes to identify the best one in terms of docking performance, which was described by its capability in terms of placing the ligand into its position in the crystal. Toward this end, we used standard precision and extra precision docking schemes, both of which are implemented in Schrodinger software.^86^ The schemes with enhanced sampling are named SP2 and XP2. On the other hand, when we used a scheme that contained both enhanced sampling and enhanced planarity, we named the methods as SP3 and XP3. We achieved more accurate results with the XP3 method, wherein the enhanced planarity of conjugated pi groups was implemented. In docking, we used crystal structures with PDB IDs: 1Z3F,^87^3FT6,^88^1D30,^89^ and 8EC1.^90^ The first two structures can be given as examples of intercalation, whereas the last two represented the groove binding. We performed docking using the OPLS3e force field^91^ and described the box dimensions to include the whole DNA present in the crystal structures studied. The molecules other than the ligand and DNA, which were present in the crystal, were removed before performing docking studies. The ionization state of the ligands and DNA was assigned at pH 7.4.

We also performed docking studies on BSA and compound **3** using the same docking scheme, namely, XP3, that we used in DNA docking. There is a couple of crystal structures pertaining to the complex of BSA with different ligands in the PDB. Among them, we used the crystal structure of naproxen-bound BSA (PDB ID: 4OR0)^92^ and took naproxen from that structure, while the structure of BSA was obtained from the crystal structure pertaining to the apo form of the protein (PDB ID: 3V03).^93^ The ionization state of the ligand and protein was assigned at pH 7.4, and the OPLS3e force field^91^ was used. Disulfide bonds were added to the protein except for Cys34 in correspondence with its physiological state.^93^ The calcium ions and water molecules that were present in the crystal structure of BSA were kept during docking calculations.

### 4.7 Cell Culture

Human cancer cell lines (MDA-MB-231, LNCaP, Caco-2) and a human healthy cell line (HEK-293) were obtained from the European Collection of Cell Cultures. The cells were incubated in a humidified incubator (37 °C, 5% CO_2_) and cultured under standard conditions in Dulbecco’s modified Eagle medium (DMEM), 10% heat-inactivated fetal bovine serum, 100 U mL^–1^ penicillin, 100 U mL^–1^ streptomycin, and 4 mM l-glutamine. Compound **3** (10 mM in DMF), clinically used cisplatin (Cipintu, 100 mg/100 mL), and etoposide (10 mM in DMF) formulation was used as stock solution in experiments. A cell culture medium was used to make further dilutions, while keeping the equivalent of DMF below 0.5% of the total volume.^94^

The detailed procedures of the cell viability inhibition assay, Annexin V/PI double-staining assay, MMP, and ROS determination studies are provided in the SI.

## Data Availability

The data underlying this study are available in the published article and its Supporting Information.

## Supporting Information Available

Synthesis and characterization (IR, ^1^H NMR) of compounds (**1** and **2**); IR spectra of compound **3**; results obtained from redocking studies; interactions formed between naproxen and BSA; and detailed experimental procedures for in vitro/in silico DNA/BSA binding studies, cell viability inhibition assay, Annexin V/PI double-staining assay, MMP, and ROS determination (PDF)
Crystallographic data (CIF)

## Supplementary Material

ao3c03355_si_001.pdf

ao3c03355_si_002.cif

## Notes

^#^ Regenerative and Restorative Medicine Research Center (REMER), Institute for Health Sciences and Technologies (SABITA), Istanbul Medipol University, 34810 Istanbul, Türkiye.

## References

[ref1] Sung H.; Ferlay J.; Siegel R. L.; Laversanne M.; Soerjomataram I.; Jemal A.; Bray F. Global cancer statistics 2020: GLOBOCAN estimates of incidence and mortality worldwide for 36 cancers in 185 countries. Ca-Cancer J. Clin. 2021, 71, 209–249. 10.3322/caac.21660. 33538338

[ref2] Innocenti F.; Ratain M. J. Update on pharmacogenetics in cancer chemotherapy. Eur. J. Cancer 2002, 38, 639–644. 10.1016/S0959-8049(01)00434-8. 11916544

[ref3] Lee W.; Lockhart A. C.; Kim R. B.; Rothenberg M. L. Cancer pharmacogenomics: powerful tools in cancer chemotherapy and drug development. Oncologist 2005, 10, 104–111. 10.1634/theoncologist.10-2-104. 15709212

[ref4] Rana M.; Faizan M. I.; Dar S. H.; Ahmad T.; Rahisuddin Design and Synthesis of Carbothioamide/Carboxamide-Based Pyrazoline Analogs as Potential Anticancer Agents: Apoptosis, Molecular Docking, ADME Assay, and DNA Binding Studies. ACS Omega 2022, 7, 22639–22656. 10.1021/acsomega.2c02033. 35811873 PMC9260921

[ref5] Mann J.; Baron A.; Opoku-Boahen Y.; Johansson E.; Parkinson G.; Kelland L. R.; Neidle S. A new class of symmetric bisbenzimidazole-based DNA minor groove-binding agents showing antitumor activity. J. Med. Chem. 2001, 44, 138–144. 10.1021/jm000297b. 11170623

[ref6] Agalave S. G.; Maujan S. R.; Pore V. S. Click chemistry: 1, 2, 3-triazoles as pharmacophores. Chem. - Asian J. 2011, 6, 2696–2718. 10.1002/asia.201100432. 21954075

[ref7] Tittal R. K.; Yadav P.; Lal K.; Kumar A.; et al. Synthesis, molecular docking and DFT studies on biologically active 1, 4-disubstituted-1, 2, 3-triazole-semicarbazone hybrid molecules. New J. Chem. 2019, 43, 8052–8058. 10.1039/C9NJ00473D.

[ref8] Struthers H.; Mindt T. L.; Schibli R. Metal chelating systems synthesized using the copper (I) catalyzed azide-alkyne cycloaddition. Dalton Trans. 2010, 39, 675–696. 10.1039/B912608B. 20066208

[ref9] Thirumurugan P.; Matosiuk D.; Jozwiak K. Click chemistry for drug development and diverse chemical–biology applications. Chem. Rev. 2013, 113, 4905–4979. 10.1021/cr200409f. 23531040

[ref10] Kallander L. S.; Lu Q.; Chen W.; Tomaszek T.; Yang G.; Tew D.; Meek T. D.; Hofmann G. A.; Schulz-Pritchard C. K.; Smith W. W.; Janson C. A.; Ryan M. D.; Zhang G.; Johanson K. O.; Kirkpatrick R. B.; Ho T. F.; Fisher P. W.; Mattern M. R.; Johnson R. K.; Hansbury M. J.; Winkler J. D.; Ward K. W.; Veber D. F.; Thompson S. K. 4-Aryl-1, 2, 3-triazole: a novel template for a reversible methionine aminopeptidase 2 inhibitor, optimized to inhibit angiogenesis in vivo. J. Med. Chem. 2005, 48, 5644–5647. 10.1021/jm050408c. 16134930

[ref11] Röhrig U. F.; Majjigapu S. R.; Grosdidier A.; Bron S.; Stroobant V.; Pilotte L.; Colau D.; Vogel P.; Van den Eynde B. J.; Zoete V.; Michielin O. Rational design of 4-aryl-1, 2, 3-triazoles for indoleamine 2, 3-dioxygenase 1 inhibition. J. Med. Chem. 2012, 55, 5270–5290. 10.1021/jm300260v. 22616902

[ref12] Krzywik J.; Nasulewicz-Goldeman A.; Mozga W.; Wietrzyk J.; Huczyński A. Novel Double-Modified Colchicine Derivatives Bearing 1, 2, 3-Triazole: Design, Synthesis, and Biological Activity Evaluation. ACS Omega 2021, 6, 26583–26600. 10.1021/acsomega.1c03948. 34661013 PMC8515607

[ref13] Sheng C.; Zhang W. New lead structures in antifungal drug discovery. Curr. Med. Chem. 2011, 18, 733–766. 10.2174/092986711794480113. 21182484

[ref14] Shlaes D. M. New β-lactam−β-lactamase inhibitor combinations in clinical development. Ann. N. Y. Acad. Sci. 2013, 1277, 105–114. 10.1111/nyas.12010. 23346860

[ref15] aPerabo F. G.; Wirger A.; Kamp S.; Lindner H.; Schmidt D. H.; Müller S. C.; Kohn E. C. Carboxyamido-triazole (CAI), a signal transduction inhibitor induces growth inhibition and apoptosis in bladder cancer cells by modulation of Bcl-2. Anticancer Res. 2004, 24, 2869–2878. 15517890

[ref16] Maurya N.; Imtiyaz K.; Rizvi M. M. A.; Khedher K. M.; Singh P.; Patel R. Comparative in vitro cytotoxicity and binding investigation of artemisinin and its biogenetic precursors with ctDNA. RSC Adv. 2020, 10, 24203–24214. 10.1039/D0RA02042G. 35516214 PMC9055135

[ref17] Rezki N.; Al-Blewi F. F.; Al-Sodies S. A.; Alnuzha A. K.; Messali M.; Ali I.; Aouad M. R. Synthesis, characterization, DNA binding, anticancer, and molecular docking studies of novel imidazolium-based ionic liquids with fluorinated phenylacetamide tethers. ACS Omega 2020, 5, 4807–4815. 10.1021/acsomega.9b03468. 32201766 PMC7081306

[ref18] Das A.; Dutta S. Binding studies of aloe-active compounds with G-quadruplex sequences. ACS Omega 2021, 6, 18344–18351. 10.1021/acsomega.1c02207. 34308065 PMC8296576

[ref19] Coury J. E.; Mcfail-Isom L.; Williams L. D.; Bottomley L. A. A novel assay for drug-DNA binding mode, affinity, and exclusion number: scanning force microscopy. Proc. Natl. Acad. Sci. 1996, 93, 12283–12286. 10.1073/pnas.93.22.12283. 8901572 PMC37982

[ref20] Espósito B. P.; Najjar R. Interactions of antitumoral platinum-group metallodrugs with albumin. Coord. Chem. Rev. 2002, 232, 137–149. 10.1016/S0010-8545(02)00049-8.

[ref21] Zhang Z.; Jin L.; Qian X.; Wei M.; Wang Y.; Wang J.; Yang Y. Y.; Xu Q.; Xu Y. T.; Liu F. Novel Bcl-2 inhibitors: discovery and mechanism study of small organic apoptosis-inducing agents. ChemBioChem 2007, 8, 113–121. 10.1002/cbic.200600305. 17139689

[ref22] Sasmal M.; Bhowmick R.; Musha Islam A. S.; Bhuiya S.; Das S.; Ali M. Domain-specific association of a phenanthrene–pyrene-based synthetic fluorescent probe with bovine serum albumin: Spectroscopic and molecular docking analysis. ACS Omega 2018, 3, 6293–6304. 10.1021/acsomega.8b00186. 31458811 PMC6644396

[ref23] Zhang Z.; Yang M.; Yi J.; Zhu Q.; Huang C.; Chen Y.; Hu W.; Chen Y.; Zhao X. Comprehensive insights into the interactions of two emerging bromophenolic DBPs with human serum albumin by multispectroscopy and molecular docking. ACS Omega 2019, 4, 563–572. 10.1021/acsomega.8b03116.

[ref24] Pal S. K.; Tew B. Y.; Lim M.; Stankavich B.; He M.; Pufall M.; Hu W.; Chen Y.; Jones J. O. Mechanistic investigation of the androgen receptor DNA-binding domain inhibitor pyrvinium. ACS Omega 2019, 4, 2472–2481. 10.1021/acsomega.8b03205. 30873507 PMC6410682

[ref25] Göktürk T.; Hökelek T.; Güp R. Synthesis, Crystal Structure and Hirshfeld Surface Analysis of Ethyl 4-(4-(2-Bromoethyl)-1H-1, 2, 3-triazol-1-yl) benzoate. Crystallogr. Rep. 2021, 66, 977–984. 10.1134/S1063774521060109.

[ref26] Şahin İ. Synthesis And Characterization Of Schiff Bases Containing 1, 2, 3-Triazole Unit: Photophysical And Acetyl Choline (Ache) Inhibitory Properties. J. Struct. Chem. 2022, 63, 1787–1796. 10.1134/S0022476622110087.

[ref27] Deswal S.; Tittal R. K.; Vikas D. G.; Lal K.; Kumar A. 5-Fluoro-1H-indole-2, 3-dione-triazoles-synthesis, biological activity, molecular docking, and DFT study. J. Mol. Struct. 2020, 1209, 127982 10.1016/j.molstruc.2020.127982.

[ref28] Bal M.; Tümer M.; Köse M. Investigation of Chemosensing and Color Properties of Schiff Base Compounds Containing a 1, 2, 3-triazole Group. J. Fluoresc. 2022, 32, 2237–2256. 10.1007/s10895-022-03007-z. 36044163

[ref29] Sharma K.; Tittal R. K.; Lal K.; Mathpati R. S. Fluorescent 7-azaindole N-linked 1, 2, 3-triazole: synthesis and study of antimicrobial, molecular docking, ADME and DFT properties. New J. Chem. 2023, 47, 9077–9086. 10.1039/D3NJ00223C.

[ref30] Paul S.; Roy P.; Saha Sardar P.; Majhi A. Design, synthesis, and biophysical studies of novel 1, 2, 3-triazole-based quinoline and coumarin compounds. ACS Omega 2019, 4, 7213–7230. 10.1021/acsomega.9b00414.

[ref31] Güngör S. A.; Köse M.; Tümer M.; Türkeş C.; Beydemir Ş. Synthesis, characterization and docking studies of benzenesulfonamide derivatives containing 1, 2, 3-triazole as potential ınhibitor of carbonic anhydrase I-II enzymes. J. Biomol. Struct. Dyn. 2022, 2159531 10.1080/07391102.2022.2159531. 36576122

[ref32] Bernstein J.; Davis R. E.; Shimoni L.; Chang N. L. Patterns in hydrogen bonding: functionality and graph set analysis in crystals. Angew. Chem., Int. Ed. 1995, 34, 1555–1573. 10.1002/anie.199515551.

[ref33] Hirshfeld F. L. Bonded-atom fragments for describing molecular charge densities. Theor. Chim. Acta 1977, 44, 129–138. 10.1007/BF00549096.

[ref34] Spackman M. A.; Jayatilaka D. Hirshfeld surface analysis. CrystEngComm 2009, 11, 19–32. 10.1039/B818330A.

[ref35] Turner M. J.; McKinnon J. J.; Wolff S. K.; Grimwood D. J.; Spackman P. R.; Jayatilaka D.; Spackman M. A. CrystalExplorer17; The University of Western Australia, 2017.

[ref36] Venkatesan P.; Thamotharan S.; Ilangovan A.; Liang H.; Sundius T. Crystal structure, Hirshfeld surfaces and DFT computation of NLO active (2E)-2-(ethoxycarbonyl)-3-[(1-methoxy-1-oxo-3-phenylpropan-2-yl) amino] prop-2-enoic acid. Spectrochim. Acta, Part A 2016, 153, 625–636. 10.1016/j.saa.2015.09.002. 26452098

[ref37] Spackman M. A.; McKinnon J. J.; Jayatilaka D. Electrostatic potentials mapped on Hirshfeld surfaces provide direct insight into intermolecular interactions in crystals. CrystEngComm 2008, 10, 377–388. 10.1039/b715227b.

[ref38] Jayatilaka D.; Grimwood D. J.; Lee A.; Lemay A.; Russel A. J.; Taylor C.; Wolff S. K.; Cassam-Chenai P.; Whitton A. TONTO—A System for Computational Chemistry, 2005. http://hirshfeldsurface.net/.

[ref39] McKinnon J. J.; Jayatilaka D.; Spackman M. A. Towards quantitative analysis of intermolecular interactions with Hirshfeld surfaces. Chem. Commun. 2007, 37, 3814–3816. 10.1039/b704980c. 18217656

[ref40] Hathwar V. R.; Sist M.; Jørgensen M. R.; Mamakhel A. H.; Wang X.; Hoffmann C. M.; Sugimoto K.; Overgaard J.; Iversen B. B. Quantitative analysis of intermolecular interactions in orthorhombic rubrene. IUCrJ 2015, 2, 563–574. 10.1107/S2052252515012130. PMC4547824 26306198

[ref41] Turner M. J.; McKinnon J. J.; Jayatilaka D.; Spackman M. A. Visualisation and characterisation of voids in crystalline materials. CrystEngComm 2011, 13, 1804–1813. 10.1039/C0CE00683A.

[ref42] Turner M. J.; Grabowsky S.; Jayatilaka D.; Spackman M. A. Accurate and efficient model energies for exploring intermolecular interactions in molecular crystals. J. Phys. Chem. Lett. 2014, 5, 4249–4255. 10.1021/jz502271c. 26273970

[ref43] Turner M. J.; Thomas S. P.; Shi M. W.; Jayatilaka D.; Spackman M. A. Energy frameworks: insights into interaction anisotropy and the mechanical properties of molecular crystals. Chem. Commun. 2015, 51, 3735–3738. 10.1039/C4CC09074H. 25525647

[ref44] Mackenzie C. F.; Spackman P. R.; Jayatilaka D.; Spackman M. A. CrystalExplorer model energies and energy frameworks: extension to metal coordination compounds, organic salts, solvates and open-shell systems. IUCrJ 2017, 4, 575–587. 10.1107/S205225251700848X. PMC5600021 28932404

[ref45] Sirajuddin M.; Ali S.; Badshah A. Drug–DNA interactions and their study by UV–Visible, fluorescence spectroscopies and cyclic voltametry. J. Photochem. Photobiol., B 2013, 124, 1–19. 10.1016/j.jphotobiol.2013.03.013. 23648795

[ref46] Dash S. P.; Panda A. K.; Pasayat S.; Dinda R.; Biswas A.; Tiekink E. R.; Mukhopadhyay S.; Bhutia S. K.; Kaminsky W.; Sinn E. Oxidovanadium (v) complexes of Aroylhydrazones incorporating heterocycles: Synthesis, characterization and study of DNA binding, photo-induced DNA cleavage and cytotoxic activities. RSC Adv. 2015, 5, 51852–51867. 10.1039/C4RA14369H.

[ref47] Şengül E. E.; Göktürk T.; Topkaya C. G.; Gup R. Synthesis, characterization and dna interaction of Cu (II) complexes with hydrazone-Schiff base ligands bearing alkyl quaternary ammonium salts. J. Chil. Chem. Soc. 2020, 65, 4754–4758. 10.4067/S0717-97072020000204754.

[ref48] Wolfe A.; Shimer G. H. Jr; Meehan T. Polycyclic aromatic hydrocarbons physically intercalate into duplex regions of denatured DNA. Biochemistry 1987, 26, 6392–6396. 10.1021/bi00394a013. 3427013

[ref49] LePecq J. B.; Paoletti C. A fluorescent complex between ethidium bromide and nucleic acids: physical–chemical characterization. J. Mol. Biol. 1967, 27, 87–106. 10.1016/0022-2836(67)90353-1. 6033613

[ref50] Mehdinia A.; Kazemi S. H.; Bathaie S. Z.; Alizadeh A.; Shamsipur M.; Mousavi M. F. Electrochemical DNA nano-biosensor for the study of spermidine–DNA interaction. J. Pharmaceut. Biomed. 2009, 49, 587–593. 10.1016/j.jpba.2008.12.035. 19186020

[ref51] Hajian R.; Tavakol M. Interaction of anticancer drug methotrexate with ds-DNA analyzed by spectroscopic and electrochemical methods. E-J. Chem. 2012, 9, 471–480. 10.1155/2012/378674.

[ref52] Mallappa M.; Gowda B. G.; Mahesh R. T. Mechanism of interaction of antibacterial drug moxifloxacin with herring sperm DNA: electrochemical and spectroscopic studies. Der Pharma Chem. 2014, 6, 398–405.

[ref53] Nehra N.; Kumar Tittal R.; Ghule V. D.; Kumar N.; Kumar Paul A.; Lal K.; Kumar A. CuAAC Mediated Synthesis of 2-HBT Linked Bioactive 1, 2, 3-Triazole Hybrids: Investigations through Fluorescence, DNA Binding, Molecular Docking, ADME Predictions and DFT Study. ChemistrySelect 2021, 6, 685–694. 10.1002/slct.202003919.

[ref54] Nehra N.; Tittal R. K.; Ghule V. D. 1, 2, 3-Triazoles of 8-Hydroxyquinoline and HBT: Synthesis and Studies (DNA Binding, Antimicrobial, Molecular Docking, ADME, and DFT). ACS Omega 2021, 6, 27089–27100. 10.1021/acsomega.1c03668. 34693129 PMC8529673

[ref55] Aouad M. R.; Almehmadi M. A.; Rezki N.; Al-blewi F. F.; Messali M.; Ali I. Design, click synthesis, anticancer screening and docking studies of novel benzothiazole-1, 2, 3-triazoles appended with some bioactive benzofused heterocycles. J. Mol. Struct. 2019, 1188, 153–164. 10.1016/j.molstruc.2019.04.005.

[ref56] Li X.; Lin Y.; Yuan Y.; Liu K.; Qian X. Novel efficient anticancer agents and DNA-intercalators of 1, 2, 3-triazol-1, 8-naphthalimides: design, synthesis, and biological activity. Tetrahedron 2011, 67, 2299–2304. 10.1016/j.tet.2011.01.063.

[ref57] Almehmadi M. A.; Aljuhani A.; Alraqa S. Y.; Ali I.; Rezki N.; Aouad M. R.; Hagar M. Design, synthesis, DNA binding, modeling, anticancer studies and DFT calculations of Schiff bases tethering benzothiazole-1, 2, 3-triazole conjugates. J. Mol. Struct. 2021, 1225, 129148 10.1016/j.molstruc.2020.129148.

[ref58] Rehman S. U.; Sarwar T.; Husain M. A.; Ishqi H. M.; Tabish M. Studying non-covalent drug–DNA interactions. Arch. Biochem. Biophys. 2015, 576, 49–60. 10.1016/j.abb.2015.03.024. 25951786

[ref59] Gokce C.; Gup R. Synthesis and characterisation of Cu (II), Ni (II), and Zn (II) complexes of furfural derived from aroylhydrazones bearing aliphatic groups and their interactions with DNA. Chem. Pap. 2013, 67, 1293–1303. 10.2478/s11696-013-0379-8.

[ref60] Gao C. Y.; Ma Z. Y.; Zhang Y. P.; Li S. T.; Gu W.; Liu X.; Tian J. L.; Xu J. Y.; Zhao J. Z.; Yan S. P. Four related mixed-ligand nickel (II) complexes: effect of steric encumbrance on the structure, DNA/BSA binding, DNA cleavage and cytotoxicity. RSC Adv. 2015, 5, 30768–30779. 10.1039/C4RA16755D.

[ref61] Munteanu A. C.; Badea M.; Olar R.; Silvestro L.; Mihaila M.; Brasoveanu L. I.; Musat M. G.; Andries A.; Uivarosi V. Cytotoxicity studies, DNA interaction and protein binding of new Al (III), Ga (III) and In (III) complexes with 5-hydroxyflavone. Appl. Organomet. Chem. 2018, 32, e4579 10.1002/aoc.4579.

[ref62] Rabbani G.; Baig M. H.; Lee E. J.; Cho W. K.; Ma J. Y.; Choi I. Biophysical study on the interaction between eperisone hydrochloride and human serum albumin using spectroscopic, calorimetric, and molecular docking analyses. Mol. Pharmaceutics 2017, 14, 1656–1665. 10.1021/acs.molpharmaceut.6b01124. 28380300

[ref63] Lakowicz J. R.; Weber G. Quenching of fluorescence by oxygen. Probe for structural fluctuations in macromolecules. Biochemistry 1973, 12, 4161–4170. 10.1021/bi00745a020. 4795686 PMC6959846

[ref64] Mitra I.; Mukherjee S.; Reddy B. V. P.; Misini B.; Misini B.; Das P.; Dasgupta S.; Dasgupta S.; Linert W.; Linert W.; Moi S. C. Synthesis, biological evaluation, substitution behaviour and DFT study of Pd (II) complexes incorporating benzimidazole derivative. New J. Chem. 2018, 42, 2574–2589. 10.1039/C7NJ05173E.

[ref65] Ramachandran E.; Raja D. S.; Bhuvanesh N. S.; Natarajan K. Mixed ligand palladium (II) complexes of 6-methoxy-2-oxo-1, 2-dihydroquinoline-3-carbaldehyde 4 N-substituted thiosemicarbazones with triphenylphosphine co-ligand: synthesis, crystal structure and biological properties. Dalton Trans. 2012, 41, 13308–13323. 10.1039/c2dt31079a. 22864662

[ref66] Patel R.; Maurya N.; Parray M. U. D.; Farooq N.; Siddique A.; Verma K. L.; Dohare N. Esterase activity and conformational changes of bovine serum albumin toward interaction with mephedrone: Spectroscopic and computational studies. J. Mol. Recognit. 2018, 31, e2734 10.1002/jmr.2734. 29920814

[ref67] Senthil Raja D.; Paramaguru G.; Bhuvanesh N. S.; Reibenspies J. H.; Renganathan R.; Natarajan K. Effect of terminal N-substitution in 2-oxo-1, 2-dihydroquinoline-3-carbaldehyde thiosemicarbazones on the mode of coordination, structure, interaction with protein, radical scavenging and cytotoxic activity of copper (II) complexes. Dalton Trans. 2011, 40, 4548–4559. 10.1039/c0dt01657h. 21431149

[ref68] Maurya N.; Maurya J. K.; Singh U. K.; Dohare R.; Zafaryab M.; Moshahid Alam Rizvi M.; Kumari M.; Patel R. In vitro cytotoxicity and interaction of noscapine with human serum albumin: Effect on structure and esterase activity of HSA. Mol. Pharmaceutics 2019, 16, 952–966. 10.1021/acs.molpharmaceut.8b00864. 30629454

[ref69] Liang J.; Cheng Y.; Han H. Study on the interaction between bovine serum albumin and CdTe quantum dots with spectroscopic techniques. J. Mol. Struct. 2008, 892, 116–120. 10.1016/j.molstruc.2008.05.005.

[ref70] Nekkanti S.; Veeramani K.; Kumari S. S.; Tokala R.; Shankaraiah N. A recyclable and water soluble copper (I)-catalyst: one-pot synthesis of 1, 4-disubstituted 1, 2, 3-triazoles and their biological evaluation. RSC Adv. 2016, 6, 103556–103566. 10.1039/C6RA22942E.

[ref71] Guo H. Y.; Chen Z. A.; Shen Q. K.; Quan Z. S. Application of triazoles in the structural modification of natural products. J. Enzyme Inhib. Med. Chem. 2021, 36, 1115–1144. 10.1080/14756366.2021.1890066. 34167422 PMC8231395

[ref72] Şahin İ.; Çeşme M.; Yüce N.; Tümer F. Discovery of new 1, 4-disubstituted 1, 2, 3-triazoles: in silico ADME profiling, molecular docking and biological evaluation studies. J. Biomol. Struct. Dyn. 2022, 41, 1988–2001. 10.1080/07391102.2022.2025905. 35057704

[ref73] Yadav P.; Lal K.; Kumar A.; Guru S. K.; Jaglan S.; Bhushan S. Green synthesis and anticancer potential of chalcone linked-1, 2, 3-triazoles. Eur. J. Med. Chem. 2017, 126, 944–953. 10.1016/j.ejmech.2016.11.030. 28011424

[ref74] Al Sheikh Ali A.; Khan D.; Naqvi A.; Al-Blewi F. F.; Rezki N.; Aouad M. R.; Hagar M. Design, synthesis, molecular modeling, anticancer studies, and density functional theory calculations of 4-(1, 2, 4-Triazol-3-ylsulfanylmethyl)-1, 2, 3-triazole derivatives. ACS Omega 2021, 6, 301–316. 10.1021/acsomega.0c04595. 33458482 PMC7807778

[ref75] Aneja B.; Azam M.; Alam S.; Perwez A.; Maguire R.; Yadava U.; Kavanagh K.; Daniliuc C. G.; Rizvi M. M. A.; Haq Q. M. R.; Abid M. Natural product-based 1, 2, 3-triazole/sulfonate analogues as potential chemotherapeutic agents for bacterial infections. ACS Omega 2018, 3, 6912–6930. 10.1021/acsomega.8b00582. 30023966 PMC6044994

[ref76] Güngör S. A.; Tümer M.; Köse M.; Erkan S. Benzaldehyde derivatives with functional propargyl groups as α-glucosidase inhibitors. J. Mol. Struct. 2020, 1206, 127780 10.1016/j.molstruc.2020.127780.

[ref77] Babin V.; Sallustrau A.; Loreau O.; Caillé F.; Goudet A.; Cahuzac H.; Del Vecchio A.; Taran F.; Audisio D. A general procedure for carbon isotope labeling of linear urea derivatives with carbon dioxide. Chem. Commun. 2021, 57, 6680–6683. 10.1039/D1CC02665H. 34132265

[ref78] Sheldrick G. M. SHELXT–Integrated space-group and crystal-structure determination. Acta Crystallogr., Sect. A: Found. Adv. 2015, 71, 3–8. 10.1107/S2053273314026370. 25537383 PMC4283466

[ref79] Sheldrick G. M. Crystal structure refinement with SHELXL. Acta Crystallogr., Sect. C: Struct. Chem. 2015, 71, 3–8. 10.1107/S2053229614024218. 25567568 PMC4294323

[ref80] Farrugia L. J. WinGX and ORTEP for Windows: an update. J. Appl. Crystallogr. 2012, 45, 849–854. 10.1107/S0021889812029111.

[ref81] Spek A. L. checkCIF validation ALERTS: what they mean and how to respond. Acta Crystallogr., Sect. E: Crystallogr. Commun. 2020, 76, 1–11. 10.1107/S2056989019016244. 31921444 PMC6944088

[ref82] Göktürk T.; Topkaya C.; Sakallı Çetin E.; Güp R. New trinuclear nickel (II) complexes as potential topoisomerase I/IIα inhibitors: in vitro DNA binding, cleavage and cytotoxicity against human cancer cell lines. Chem. Pap. 2022, 76, 2093–2109. 10.1007/s11696-021-02005-y.

[ref83] Loganathan R.; Ganeshpandian M.; Bhuvanesh N. S.; Palaniandavar M.; Muruganantham A.; Ghosh S. K.; Riyasdeen A.; Akbarsha M. A. DNA and protein binding, double-strand DNA cleavage and cytotoxicity of mixed ligand copper (II) complexes of the antibacterial drug nalidixic acid. J. Inorg. Biochem. 2017, 174, 1–13. 10.1016/j.jinorgbio.2017.05.001. 28551479

[ref84] Bellam R.; Jaganyi D.; Robinson R. S. Heterodinuclear Ru–Pt Complexes Bridged with 2, 3-Bis (pyridyl) pyrazinyl Ligands: Studies on Kinetics, Deoxyribonucleic Acid/Bovine Serum Albumin Binding and Cleavage, In Vitro Cytotoxicity, and In Vivo Toxicity on Zebrafish Embryo Activities. ACS Omega 2022, 7, 26226–26245. 10.1021/acsomega.2c01845. 35936428 PMC9352169

[ref85] Parsekar S. U.; Velankanni P.; Sridhar S.; Haldar P.; Mate N. A.; Banerjee A.; Antharjanam P. K. S.; Koley A. P.; Kumar M. Protein binding studies with human serum albumin, molecular docking and in vitro cytotoxicity studies using HeLa cervical carcinoma cells of Cu (II)/Zn (II) complexes containing a carbohydrazone ligand. Dalton Trans. 2020, 49, 2947–2965. 10.1039/C9DT04656A. 32073070

[ref86] Friesner R. A.; Murphy R. B.; Repasky M. P.; Frye L. L.; Greenwood J. R.; Halgren T. A.; Sanschagrin P. C.; Mainz D. T. Extra Precision Glide: Docking and Scoring Incorporating a Model of Hydrophobic Enclosure for Protein-Ligand Complexes. J. Med. Chem. 2006, 49, 6177–6196. 10.1021/jm051256o. 17034125

[ref87] Canals A.; Purciolas M.; Aymamí J.; Coll M. The anticancer agent ellipticine unwinds DNA by intercalative binding in an orientation parallel to base pairs. Acta Crystallogr., Sect. D: Biol. Crystallogr. 2005, 61, 1009–1012. 10.1107/S0907444905015404. 15983425

[ref88] Maehigashi T.; Persil O.; Hud N. V.; Williams L. D. Crystal structure of proflavine in complex with a DNA hexamer duplex. 2009, to be published.

[ref89] Larsen T. A.; Goodsell D. S.; Cascio D.; Grzeskowiak K.; Dickerson R. E. The structure of DAPI bound to DNA. J. Biomol. Struct. Dyn. 1989, 7, 477–491. 10.1080/07391102.1989.10508505. 2627296

[ref90] Ogbonna E. N.; Paul A.; Farahat A. A.; Terrell J. R.; Mineva E.; Ogbonna V.; Boykin D. W.; Wilson W. D. X-ray Structure Characterization of the Selective Recognition of AT Base Pair Sequences. ACS Bio Med. Chem. Au 2023, 5658 10.1021/acsbiomedchemau.3c00002. PMC10436263 37599788

[ref91] Roos K.; Wu C.; Damm W.; Reboul M.; Stevenson J. M.; Lu C.; Dahlgren M. K.; Mondal S.; Chen W.; Wang L.; Abel R.; Friesner R. A.; Harder E. D. OPLS3e: Extending Force Field Coverage for Drug-Like Small Molecules. J. Chem. Theory Comput. 2019, 15, 1863–1874. 10.1021/acs.jctc.8b01026. 30768902

[ref92] Bujacz A.; Zielinski K.; Sekula B. Structural studies of bovine, equine, and leporine serum albumin complexes with naproxen. Proteins 2014, 82, 2199–2208. 10.1002/prot.24583. 24753230

[ref93] Majorek K. A.; Porebski P. J.; Dayal A.; Zimmerman M. D.; Jablonska K.; Stewart A. J.; Chruszcz M.; Minor W. Structural and immunologic characterization of bovine, horse, and rabbit serum albumins. Mol. Immunol. 2012, 52, 174–182. 10.1016/j.molimm.2012.05.011. 22677715 PMC3401331

[ref94] Mosmann T. Rapid colorimetric assay for cellular growth and survival: application to proliferation and cytotoxicity assays. J. Immunol. Methods 1983, 65, 55–63. 10.1016/0022-1759(83)90303-4. 6606682

