# Supplementary material for: Synthesis, Structural Investigations, DNA/BSA Interactions, Molecular Docking Studies, and Anticancer Activity of a New 1,4-Disubstituted 1,2,3-Triazole Derivative
Source: ACS Omega. 2023 Aug 25;8(35):31839–56. doi: 10.1021/acsomega.3c03355 (PMC10483525; doi:10.1021/acsomega.3c03355)
Supplement: Supplementary file 1 — ao3c03355_si_001.pdf [file ao3c03355_si_001.pdf]

Supplementary Information

Synthesis, Structural Investigations, DNA/BSA Interactions, Molecular Docking Studies and Anticancer Activity of a New 1,4-disubstituted 1,2,3-triazole Derivative

Tolga GÖKTÜRK[1*], Esin Sakallı ÇETİN[2], Tuncer HÖKELEK[3], Hanife PEKEL[4†], Özge ŞENSOY[5†], Ebru Nur AKSU[2], Ramazan GÜP[1]

[1]Department of Chemistry, Muğla Sıtkı Koçman University, 48000 Muğla, Türkiye

[2]Department of Medical Biology, Muğla Sıtkı Koçman University, 48000 Muğla, Türkiye

[3]Department of Physics, Hacettepe University, 06800 Ankara, Türkiye.

[4]Department of Pharmacy Services, Vocational School of Health Services, Istanbul Medipol University, Istanbul, Türkiye

[5]Department of Computer Engineering, Istanbul Medipol University, 34000 Istanbul, Türkiye

[†]Regenerative and Restorative Medicine Research Center (REMER), Institute for Health Sciences and Technologies (SABITA), Istanbul Medipol University, Türkiye

**Corresponding author email: tolgagokturk@mu.edu.tr*

Table of Content

1. Synthesis of 2-hydroxy-4-(prop-2-yn-1-yloxy)benzaldehyde ……………………..............S2
2. Synthesis of 4-azido-1,2-dichlorobenzene ……………..................................….............S3
3. FT-IR Spectrum of Compound 3…………………...S5
4. DNA Binding Studies………………………………………….....................................S7
5. BSA Binding Studies……………………………………..……………………………...S8
6. In Silico DNA/BSA Binding Studies……………………………...………………….S9
7. Cell Viability Inhibition Assay……………………………………...……………………..S9
8. Annexin-V/PI double-staining Assay……………………………......……………...S10
9. MMP Determination………………………….....…...……………...……………...S10
10. ROS Determination…………………………...………………………………….....S10

1. Synthesis of 2-hydroxy-4-(prop-2-yn-1-yloxy)benzaldehyde

2,4-dihydroxybenzaldeyhde and potassium bicarbonate (10 mmol) were suspended in acetonitrile (30 mL) and refluxed for 45 min. Propargyl bromide (15 mmol) was added and the reaction mixture was refluxed for 30 h. When the completion of the reaction was confirmed by TLC, the reaction mixture was filtered while hot and the solvent was removed by rotary evaporation. The remaining black solid in the flask was extracted three times with chloroform and 3M HCl solution. After extraction, the combined organic phases were dried with anhydrous $NaSO_4$, filtered and a brown solid was obtained after removing the solvent. The product was purified by silica gel column chromatography by eluting with a mixture of 10% ethyl acetate in n-hexane. Evaporation of the purified extract yielded pure product.

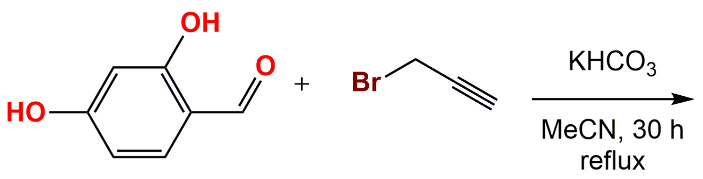

Scheme S1. Synthesis of 2-hydroxy-4-(prop-2-yn-1-yloxy)benzaldehyde

Synthesis of 2-hydroxy-4-(prop-2-yn-1-yloxy)benzaldehyde (1): $C_{10}H_8O_3$, 176.17 g/mol. **Yield:** 47%. **Color:** White. **Melting point:** 72-74 °C. **FTIR** (ν, cm^{-1}): 3239 (OH), 2839 (C-H), 2128 (-C≡CH), 1634 (C=O), 1453 (C=C), 1360 (C-O), ^{1}**H NMR** (300 MHz, $CDCl_3$) δ (ppm): 11.47 (s, 1H), 9.76 (s, 1H), 7.48 (d, *J* = 8.7 Hz, 1H), 6.63 (dd, *J* = 8.7, 2.4 Hz, 1H), 6.55 (d, *J* = 2.4 Hz, 1H), 4.76 (d, *J* = 2.4 Hz, 2H), 2.60 (t, *J* = 2.4 Hz, 1H).

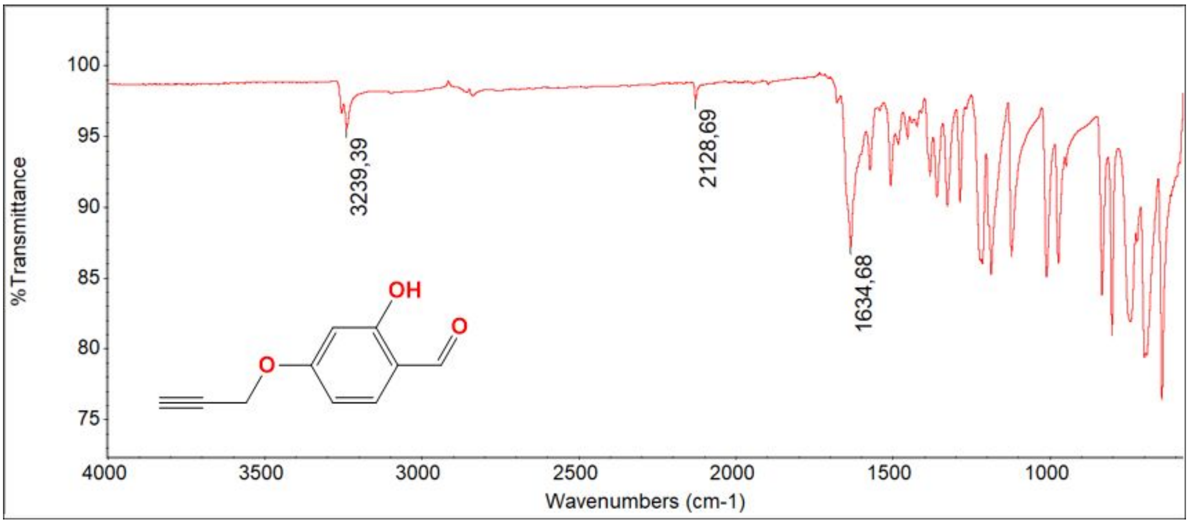

Figure S1. FTIR spectra of 2-hydroxy-4-(prop-2-yn-1-yloxy)benzaldehyde

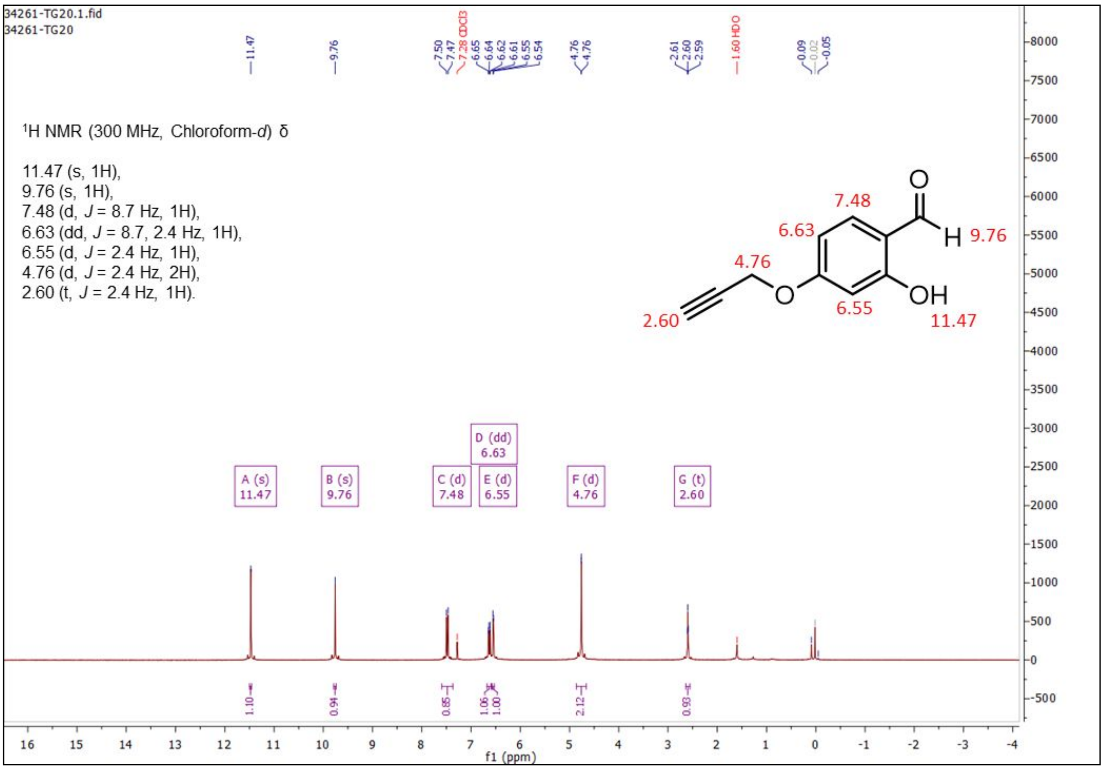

Figure S2. ^{1}H-NMR spectra of 2-hydroxy-4-(prop-2-yn-1-yloxy)benzaldehyde

2. Synthesis of 4-azido-1,2-dichlorobenzene

A solution of 3,4-dichloroaniline (10 mmol) in HCl 1.2 M (4 mL/mmol) was cooled to 0°C in an ice bath. To this stirred mixture was added a solution of $NaNO_2$ (12 mmol) in water. The solution was stirred for 30 min at 0 °C and a solution of NaN_3 (15 mmol) in water was added dropwise. The resulting solution was stirred at room temperature for 1 hour and extracted twice with ethyl acetate. The combined organic layers were dried over $MgSO_4$, filtrated and concentrated under vacuum.

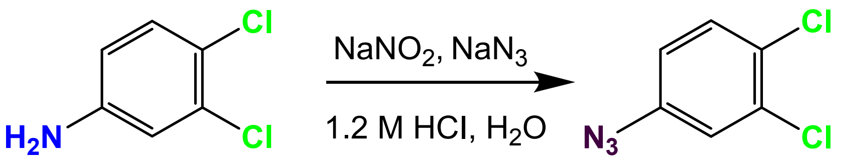

Scheme S2. Synthesis of 4-azido-1,2-dichlorobenzene

4-azido-1,2-dichlorobenzene: $C_6H_3Cl_2N_3$, 188.01 g/mol. **Yield:** 82%. **Color:** Brown. **FTIR** (ν, cm^{-1}): 2102 (N_3). **^{1}H NMR** (300 MHz, $CDCl_3$) δ (ppm): 7.41 (dq, J = 8.7, 0.5 Hz, 1H), 7.24 – 7.02 (m, 1H), 6.88 (ddt, J = 8.7, 2.6, 0.5 Hz, 1H).

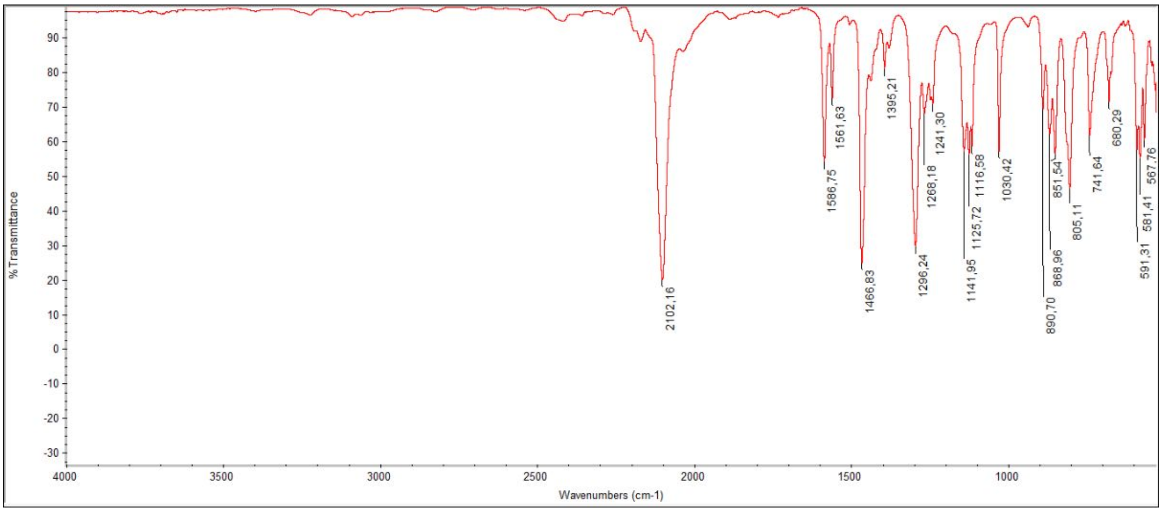

Figure S3. FTIR spectra of 4-azido-1,2-dichlorobenzene

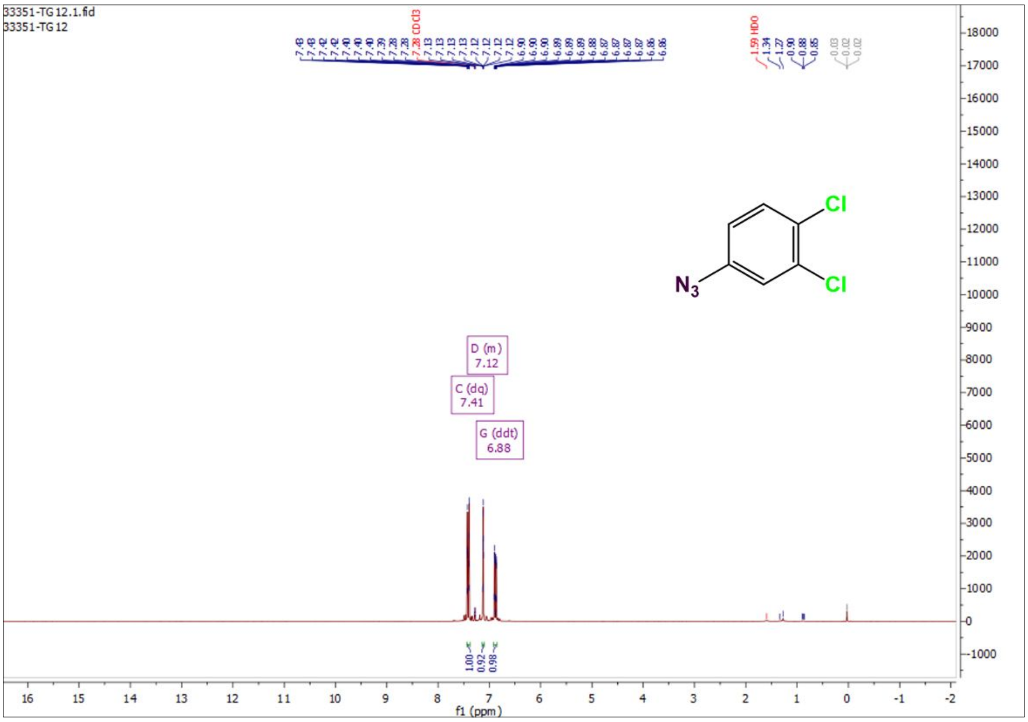

Figure S4. ^{1}H NMR spectra of 4-azido-1,2-dichlorobenzene

3. FT-IR spectrum of compound 3

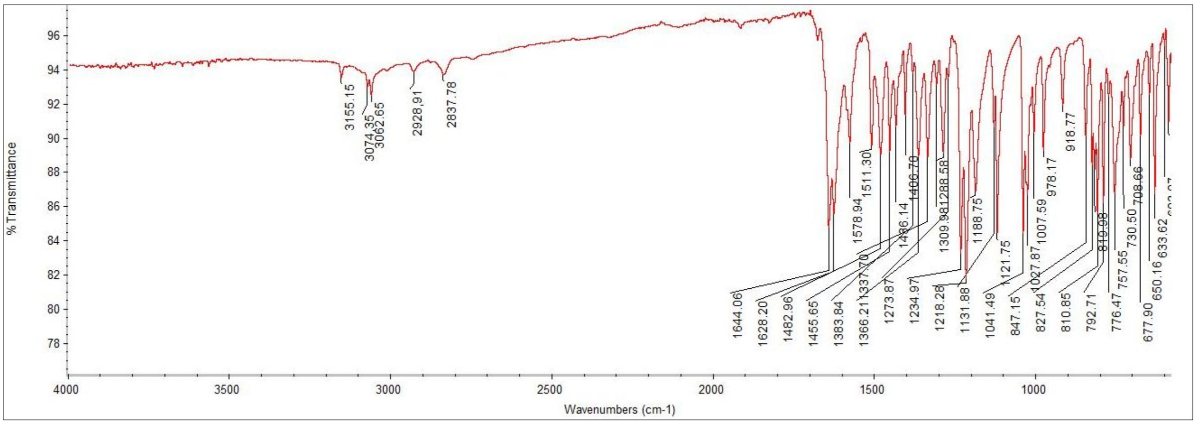

Figure S5. FT-IR spectrum of compound **3**

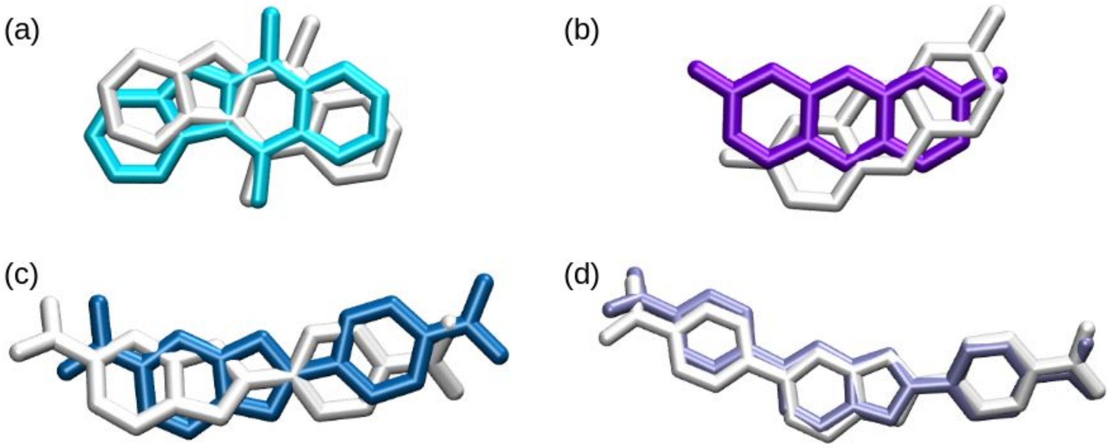

Figure S6. Presentation of the results obtained from docking studies performed with crystal structures with PDB IDs: (a) 1Z3F, (b) 3FT6, (c) 1D30, (d) 8EC1. In each panel, white color corresponds to the pose obtained by docking studies using the XP3 method.

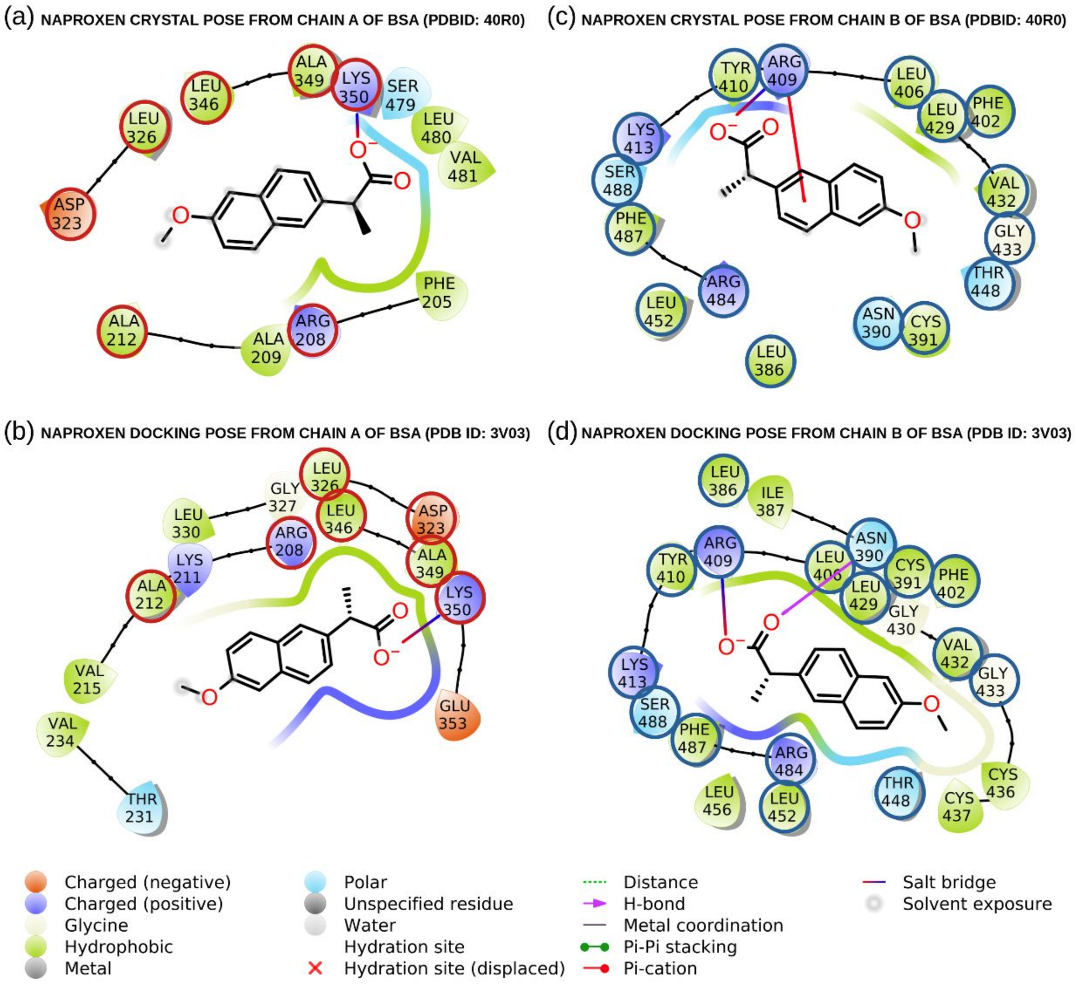

Figure S7. The interactions formed between naproxen and BSA. To present the similarity of binding region between crystal and docking poses of naproxen, contributing amino acids are shown in red and blue circles for chain A and chain B of BSA, respectively.

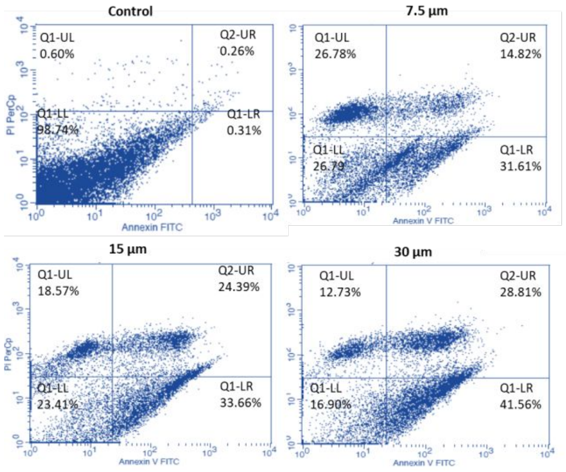

Figure S8. Annexin V and PI staining of untreated Caco2 cells and cells treated for 48 h with compound 3 at different drug doses (7.5, 15 and 30 µM). Viable cells are Annexin- and PI-. Necrotic cells are Annexin- and PI+. Early apoptotic cells are Annexin + and PI-. Late apoptotic cells are Annexin + and PI +.

4. DNA Binding Studies

Absorption spectral studies

DNA binding studies have been performed by UV–vis spectroscopic titration according to the previously reported literature with slight modifications. [1-3] Titrations were performed using buffer containing 10 mM Tris-HCI, 50 mM NaCl and this buffer was adjusted to pH 7.6 at room temperature. Stock solution of compound was prepared in 1% (V/V) DMF and 99% (V/V) Tris-HCI buffer. DNA stock solution was prepared by dissolving the commercially purchased CT-DNA in Tris-HCI buffer at 4 °C and used freshly. A solution of CT-DNA in the buffer gave a ratio of UV absorbance at 260 and 280 nm of ca. 1.8–1.9:1, indicating that the DNA was sufficiently free of protein. The DNA concentration per nucleotide was determined by absorption spectroscopy using the molar absorption coefficient (6600 M^{-1} cm^{-1}) at 260 nm. [4]

The absorption titrations were carried out by adding increasing amounts of DNA (0-100 μM) to a solution of the compound at a fixed concentration (50 μM) contained in a quartz cell. The absorption spectra were obtained by adding the equal amount of CT-DNA to both reference and sample solutions to eliminate the absorbance of CT-DNA. After each addition, the reaction mixture was allowed to incubate for 5 minutes before the absorption spectrum was recorded. The intrinsic binding constant K_b of compound was calculated by using Wolfe-Shimmer equation [5],

$$\frac{[DNA]}{(\varepsilon a - \varepsilon f)} = \frac{[DNA]}{\varepsilon b - \varepsilon f} + \frac{1}{Kb(\varepsilon b - \varepsilon f)}$$

Where [DNA] is the concentration of DNA in base pairs, ε_a, ε_f, and ε_b correspond to A_{obs}/[compound], the extinction coefficient for the free compounds, and the extinction coefficient for the compounds fully bound with DNA. In plot of [DNA] / ($\varepsilon_a - \varepsilon_f$) versus [DNA], the intrinsic binding constant K_b is given by the ratio of the slope to y-intercept. The standard Gibbs free (ΔG) of compound bound to CT-DNA was obtained using the Van't Hoff equation.

$$\Delta G = - RT \ln K_b$$

Fluorescence spectral studies

The fluorescence quenching experiments were performed for additional support for binding of compound to CT-DNA. ethidium bromide (EB) is a well-known fluorescent probe for DNA

moiety and used in evaluation of the interaction mode between a compound and CT-DNA. [6] In buffer solution, EB shows low fluorescence intensity, whereas the fluorescence intensity dramatically enhanced by treatment of EB (5 μM) with CT-DNA (25 μM) for 2 h at 24 °C. Further, the compound in different amounts of concentrations (0–150 μM) were added to the EB+CT-DNA solution. The fluorescence spectra of 200 μL/well solutions in a 96-well plate were recorded with 510 nm excitation and the emission spectra of constant [EB+CT-DNA] with varying the compound concentration were recorded (λ_{em} =500–700). Before recording the spectra, the solutions were thoroughly mixed and incubated for 10 minutes at room temperature. The quenching efficiency of the compound was analysed using the Stern-Volmer equation.

$$I_o/I = 1 + K_{sv}[Q] = 1 + k_q\tau_0[Q]$$

where I_o and I are the emission intensities of CT-DNA+EB complex in the absence and each addition of compound, respectively and [Q] is the concentration of quencher. The Stern-Volmer (quenching) constant, K_{sv} was determined from the slope of the linear plot of I_o/I versus [Q]. To have an insight into the kinetics of the competitive binding process, the bimolecular quenching rate constant, k_q values were also computed using the Stern-Volmer equation.

$$K_{SV} = k_q\tau_0$$

where τ_0 is the average fluorescence lifetime of the CT-DNA+EB complex in the absence of the quencher and its value is 23 nanoseconds at room temperature.[7] Scatchard plots also gave the binding constant K_{bin} as determined from the fluorescence titration using Scatchard equation.

$$\log(I_o - I) / I = \log K_{bin} + n \log[Q]$$

where n is the number of binding sites per nucleotide. [8]

5. BSA binding Studies

Fluorescence Spectral Studies

Quenching of fluorescence emission of BSA by compound was performed in order to determine its binding constant. Stock concentration of BSA (5 μM) was prepared in 10 mM Tris- HCl buffer at pH = 7.4. The emission spectra of 5 μM BSA solutions in the absence and presence of compound of concentration ranging from 0 to 8 μM were acquired at room temperature. The

emission changes were recorded in the λ_{em} range of 300 to 450 nm, with the excitation wavelength set at 280 nm. Each spectrum was recorded after an incubation time of 10 minutes. The quenching efficiency of the compound was calculated using the Stern-Volmer equation as discussed above. The Stern-Volmer (quenching) constant, K_{sv} was determined from the slope of the linear plot of I_o/I versus [Q]. To have an insight into the kinetics of the competitive binding process, the bimolecular quenching rate constant, k_q values were also computed using the same Stern Volmer equation, where τ_0 is the average fluorescence lifetime of the BSA alone is 10 nanoseconds. [9] Scatchard plots also gave the binding constant K_{bin} as determined from the fluorescence titration using Scatchard equation. [10] The standard Gibbs free (ΔG) of compound bound to BSA was obtained using the Van't Hoff equation ($\Delta G = - RT \ln K_{bin}$)

6. In Silico DNA/BSA Binding Studies

Table S1. Comparison of the docking schemes used in the study. The RMSD values obtained by docking the DNA with its crystal ligands.

		RMSD (Å)			
PDBID		1Z3F	3FT6	1D30	8EC1
METHOD	SP1	5.98	5.93	8.29	10.59
	SP2	5.98	1.93	8.27	10.58
	SP3	5.98	1.92	8.31	1.1
	XP1	1.24	1.93	2.16	6.34
	XP2	1.24	1.93	2.16	6.34
	XP3	0.97	1.91	1.62	1.52

7. Cell Viability Inhibition Assay

MDA-MB-231, LNCaP, Caco-2 and HEK-293 cells ($5x10^3$ per well) seeded in 96-well plates to assess cell viability by the 3-[4,5-dimethylthiazol-2-yl]-2,5-diphenyltetrazolium bromide (MTT) assay for 24 h at 37°C. Then cells were treated with compound 3 at different concentrations (3-100 µM) at 100µl/well. In parallel, the traditional anticancer agent cisplatin and etoposid was used as positive controls under identical conditions. After 24, 48 and 72 h incubation, 5 mg/ml MTT solution (20 µl/well) was added and cultured for another 4 h. Then, the supernatant was discarded and dimethyl sulfoxide was added (100 µl/well). The absorbance (A) was measured spectrophotometrically at 540 nm by a Microplate Reader (SpectraMax i3x). A graph was plotted between the concentrations of the extract versus the percent viability. The

concentration of the samples that inhibits growth of 50 % of the cells (IC50 values) was calculated from graph. All assays were performed in three replicates per concentration level. [11]

8. Annexin-V/PI Double-Staining Assay

Caco-2 ($5x10^5$ per well) were seeded in 6-well culture plates in medium and incubated for 24 h at 37°C. Then cells were either untreated or treated with the compound **3** (7.5, 15, 30 µM) for additional 48 h. The cells were harvested and resuspended in 400 µl of 1× binding buffer containing Annexin V-FITC (5 µl) and PI (5 µl), and cells were incubated for 15 min at RT (25°C) in the dark. The results were evaluated immediately through flow cytometry (BD Biosciences, San Jose, CA, USA) within 1 h using Annexin V-FITC staining kit (BD Biosciences, San Jose, CA, USA) according to the manufacturer's instructions

9. MMP Determination

Briefly, Caco-2 (5x105 per well) were seeded in 6-well plates for 24 h and either untreated or treated with a various concentrations of compound 3 (7.5, 15, 30 µM) for addional 48 h. Following treatment, cells were harvested and were incubated with 50 nM BD PharmingenTM MitoStatus TMRE for 30 min at 37°C, 5% CO_2, followed by resuspending twice with pre-warmed PBS (2000 rpm×3 min). Quantitative changes in mitochondrial membrane potential (MMP) at the early stage of cell apoptosis were measured using BD PharmingenTM MitoStatus TMRE (BD Biosciences, San Jose, CA, USA) and detected by flow cytometry (BD Biosciences, San Jose, CA, USA)

10. ROS Determination

The intracellular ROS generation was evaluated using The Total Reactive Oxygen Species (ROS) Assay Kit. Caco2 cells ($5x10^5$ per well) were seeded in 6-well plates and were incubated for 24 h in a 37°C incubator with 5% CO_2. Then cells were treated with compound 3 (7.5, 15, 30 µM) for 48 h to induce production of ROS. Cells were harvested and incubated with 10 µM H2DCFDA for 30 min at 37°C incubator with 5% CO_2, followed by resuspending three times with pre-warmed HBSS (2000 rpm×3 min). Total ROS levels in live cells were measured using a flow cytometry (BD Biosciences, San Jose, CA, USA).

References:

[1] Topkaya, C. G., Göktürk, T., Hökelek, T., Çetin, E. S., Kincal, S., & Güp, R. (2022). In vitro DNA interaction, topoisomerase I/II Inhibition and cytotoxic properties of polymeric copper (II) complex bridged with perchlorate ion containing N4-type schiff base ligand. Journal of Molecular Structure, 1266, 133453.

[2] Gökçe, C., & Gup, R. (2013). Synthesis, characterization and DNA interaction of new copper (II) complexes of Schiff base-aroylhydrazones bearing naphthalene ring. Journal of Photochemistry and Photobiology B: Biology, 122, 15-23.

[3] Göktürk, T., Topkaya, C., Sakallı Çetin, E., & Güp, R. (2022). New trinuclear nickel (II) complexes as potential topoisomerase I/IIα inhibitors: in vitro DNA binding, cleavage and cytotoxicity against human cancer cell lines. Chemical Papers, 76(4), 2093-2109.

[4] Marmur, J. (1961). A procedure for the isolation of deoxyribonucleic acid from micro-organisms. Journal of molecular biology, 3(2), 208-IN1.

[5] Pyle, A. M., Rehmann, J. P., Meshoyrer, R., Kumar, C. V., Turro, N. J., & Barton, J. K. (1989). Mixed-ligand complexes of ruthenium (II): factors governing binding to DNA. Journal of the American Chemical Society, 111(8), 3051-3058.

[6] Loganathan, R., Ganeshpandian, M., Bhuvanesh, N. S., Palaniandavar, M., Muruganantham, A., Ghosh, S. K., Riyasdeen, A., & Akbarsha, M. A. (2017). DNA and protein binding, double-strand DNA cleavage and cytotoxicity of mixed ligand copper (II) complexes of the antibacterial drug nalidixic acid. Journal of inorganic biochemistry, 174, 1-13.

[7] Heller, D. P., & Greenstock, C. L. (1994). Fluorescence lifetime analysis of DNA intercalated ethidium bromide and quenching by free dye. Biophysical chemistry, 50(3), 305-312.

[8] Bellam, R., Jaganyi, D., & Robinson, R. S. (2022). Heterodinuclear Ru–Pt Complexes Bridged with 2, 3-Bis (pyridyl) pyrazinyl Ligands: Studies on Kinetics, Deoxyribonucleic Acid/Bovine Serum Albumin Binding and Cleavage, In Vitro Cytotoxicity, and In Vivo Toxicity on Zebrafish Embryo Activities. ACS omega, 7(30), 26226-26245.

[9] Schägger, H., & Von Jagow, G. (1987). Tricine-sodium dodecyl sulfate-polyacrylamide gel electrophoresis for the separation of proteins in the range from 1 to 100 kDa. Analytical biochemistry, 166(2), 368-379.

[10] Parsekar, S. U., Velankanni, P., Sridhar, S., Haldar, P., Mate, N. A., Banerjee, A., Antharjanam, P. K., Koley, A. P. & Kumar, M. (2020). Protein binding studies with human serum albumin, molecular docking and in vitro cytotoxicity studies using HeLa cervical

carcinoma cells of Cu (II)/Zn (II) complexes containing a carbohydrazone ligand. Dalton Transactions, 49(9), 2947-2965.
[11] Mosmann, T. (1983). Rapid colorimetric assay for cellular growth and survival: application to proliferation and cytotoxicity assays. J. Immunol. Methods, 65:55–63.
